# The Histone H1-Like Protein AlgP Facilitates Even Spacing of Polyphosphate Granules in Pseudomonas aeruginosa

**DOI:** 10.1128/mbio.02463-21

**Published:** 2022-04-18

**Authors:** Ravi Chawla, Steven Klupt, Vadim Patsalo, James R. Williamson, Lisa R. Racki

**Affiliations:** a Department of Integrative Structural and Computational Biology, Scripps Research, La Jolla, California, USA; b Department of Immunology and Microbiology, Scripps Research, La Jolla, California, USA; University of California, Berkeley

**Keywords:** AlgP, polyphosphate, pseudomonas aeruginosa, cell biology, starvation

## Abstract

Synthesis of polyphosphate (polyP) is an ancient and universal stress and starvation response in bacteria. In many bacteria, polyP chains come together to form granular superstructures within cells. Some species appear to regulate polyP granule subcellular organization. Despite the critical role of polyP in starvation fitness, the composition of these structures, mechanism(s) underpinning their organization, and functional significance of such organization are poorly understood. We previously determined that granules become transiently evenly spaced on the cell’s long axis during nitrogen starvation in the opportunistic human pathogen Pseudomonas aeruginosa. Here, we developed a granule-enrichment protocol to screen for polyP granule-localizing proteins. We identified AlgP as a protein that associates with polyP granules. We further discovered that AlgP is required for the even spacing of polyP granules. AlgP is a DNA-binding protein with a 154 amino acid C-terminal domain enriched in “KPAA” repeats and variants of this repeat, with an overall sequence composition similar to the C-terminal tail of eukaryotic histone H1. Granule size, number, and spacing are significantly perturbed in the absence of AlgP, or when AlgP is truncated to remove the C-terminus. The *ΔalgP* and *algPΔ*CTD mutants have fewer, larger granules. We speculate that AlgP may contribute to spacing by tethering polyP granules to the chromosome, thereby inhibiting fusion with neighboring granules. Our discovery that AlgP facilitates granule spacing allows us for the first time to directly uncouple granule biogenesis from even spacing, and will inform future efforts to explore the functional significance of granule organization on fitness during starvation.

## INTRODUCTION

In response to diverse starvation cues, bacteria almost universally do a curious thing: They spend ATP to make an inorganic polymer of phosphoryl groups, polyphosphate (polyP). The ability to make polyP has been shown to be important for fitness during starvation in evolutionarily disparate bacterial species, but the molecular mechanism(s) underpinning these effects has remained mysterious (1, 2). A significant challenge to understanding how polyP exerts its pleiotropic effects on physiology has been the small number of validated polyP-binding proteins identified in bacteria. Aside from the polyP biosynthetic machinery (kinase families Ppk1 and Ppk2) and a phosphatase (Ppx), only a few proteins are known to functionally interact with polyP in bacteria (1, 3). These include enzymes that have been shown *in vitro* to be able to use polyP as a substrate for phosphorylation reactions, such as NAD kinases and glucokinases (1, 3). In addition, several proteins containing a positively-charged **C**onserved **H**istidine **α**-helical **D**omain (CHAD), have been shown by microscopy to localize to polyP granules (4, 5). It may be that the specificity of binding to polyP is mediated by cooperative assembly of multicomponent protein complexes, making it difficult to understand polyP binding through binary interactions. Because polyP forms granular superstructures in many bacteria, co-localization studies can serve as a powerful method to identify true polyP-interacting proteins.

We and others have observed that polyP granules are organized within cells, raising the possibility that the subcellular localization of these structures is a regulated process (6–9). An important motivation for characterizing the polyP granule proteome is therefore to identify not just polyP-utilizing proteins, but those that specifically contribute to the formation and organization of these structures. In Pseudomonas aeruginosa, polyP granules become transiently evenly spaced on the long axis of the cell during nitrogen starvation (10). In addition to the even-spacing phenomenon, a notable structural feature of polyP granules is that they form in the nucleoid region of the cell, where the chromosome resides. PolyP granules appear to affect chromosome function, as P. aeruginosa cells that cannot make polyP are deficient in exiting the cell cycle during starvation, and these cells also have an elevated SOS DNA damage response (10). PolyP also affects cell cycle progression in Caulobacter crescentus (11). Moreover, in C. crescentus, it has been shown that disrupting cell cycle progression can in turn alter granule organization (6). Recently polyP has been suggested to interact with the nucleoid associated protein (NAP) Hfq in Escherichia coli and promote Hfq-mediated transcriptional silencing (12). Together, these observations indicate that the polyP polymer and polyP granules are structurally and functionally linked to the chromosome in diverse bacterial species. Much remains to be explored about how and why polyP is connected to bacterial chromatin, but the proteome of polyP granules may provide clues.

In this study, our goal was to develop a polyP granule enrichment protocol in P. aeruginosa strain UCBPP-PA14 (herein referred to as just “PA14”) as a screening tool to characterize the polyP granule associated proteome. We then validated our protocol by confirming that one of our candidate proteins from this screen, AlgP, indeed localizes to granules. AlgP is a putative DNA binding protein that has a predicted intrinsically disordered C-terminal domain containing tandem repeats of KPAA residues (13, 14). Prior studies have demonstrated that this C-terminal domain is a mutational hot spot in clinical isolates of P. aeruginosa from the lungs of cystic fibrosis (CF) patients, raising the possibility that *algP* may be under selective pressure in chronic infections (15, 16). The C-terminal domain of AlgP has also previously been noted for its similarities to eukaryotic histone H1, due to its enrichment in lysine, alanine and proline residues, intrinsic disorder, and the ability of C-terminally-derived peptides to bind to DNA (14, 15, 17, 18). We found that the C-terminus of AlgP is required for granule spacing but that AlgP does not play a role in efficient cell cycle exit during nitrogen starvation. Our proteomic screen represents the first step in determining the interactome of polyP in the opportunistic human pathogen P. aeruginosa, and hopefully other bacteria in the future.

## RESULTS

### PolyP granule enrichment and proteomic analysis.

**FIG 1:**
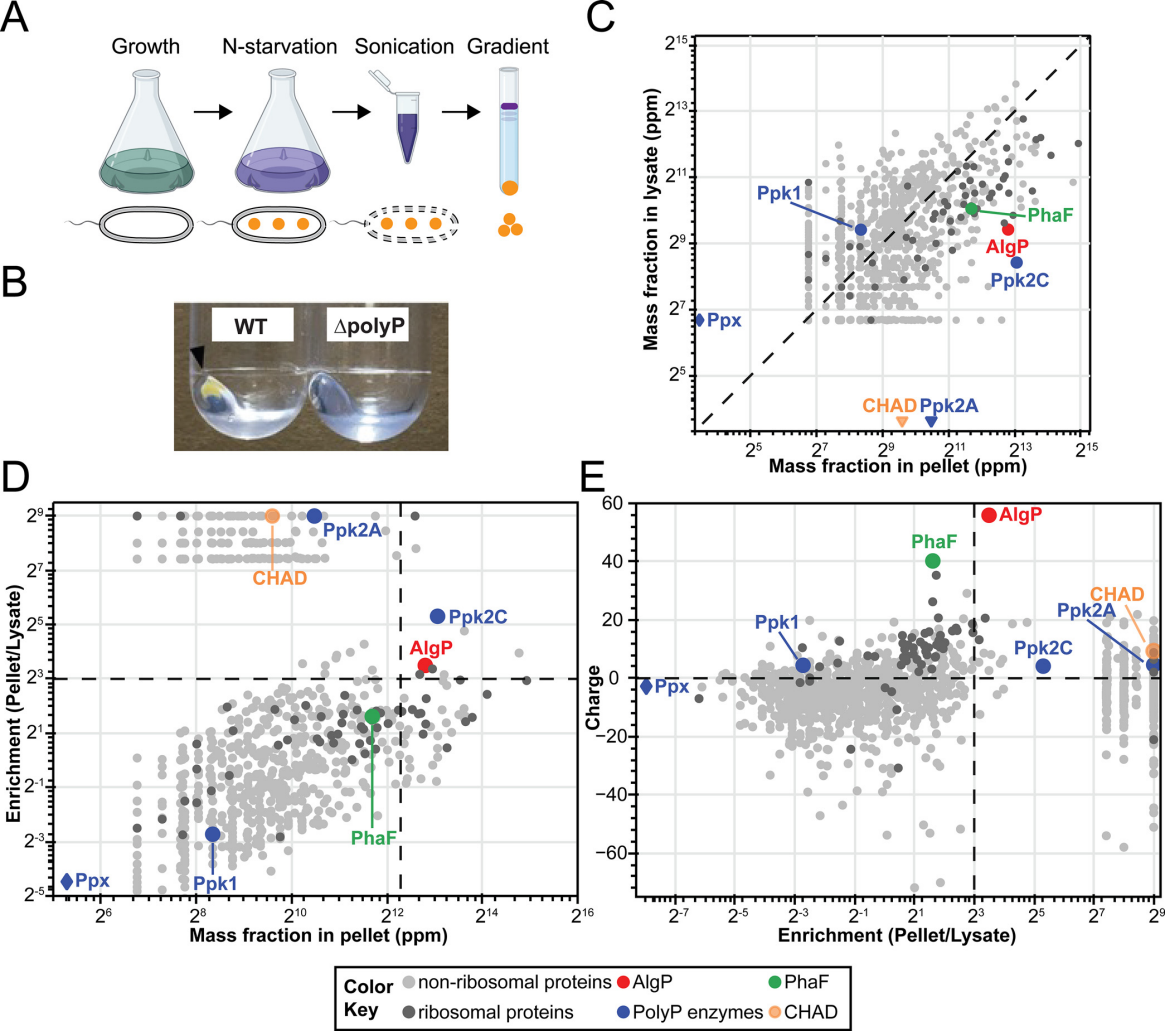
Workflow for isolation of polyphosphate granules from starved P. aeruginosa cells and proteomic analysis. (A) The workflow of granule isolation as described in the Materials and Methods section. (B) The ultracentrifugation of a lysate from wild type Pseudomonas aeruginosa *PA14* cells in a Percoll gradient solution generates a “pellet” fraction, which is not seen with a lysate from the ΔpolyP strain. This panel shows a zoomed-in view of the Percoll-encased pellet after discarding the Percoll gradient solution. (C to E) Mass spectrometry analysis of the pellet fractions from three independent experiments. (C) Absolute mass fraction of proteins in the “pellet” and “lysate,” obtained from spectral counting (see Materials and Methods and Fig. S1) is shown in parts per million. The dashed diagonal corresponds to a line of equal abundance in the pellet and lysate fraction. The data points below the diagonal line correspond to proteins in higher abundance in the pellet fraction than in the lysate; diamond and inverted triangle symbol marks the proteins identified in “lysate-only” and “pellet-only” fractions. (D) The enrichment of proteins in the pellet plotted against the abundance of proteins in the pellet. The cut-off lines for enrichment is 8, (2^3^, log_2_ fold change =3) and for abundance is 5,000 ppm. The highly abundant and enriched proteins in the pellets are, therefore, located in the upper right quadrant. (E) The calculated charge at neutral pH for proteins identified in the proteomics plotted against the fold enrichment (negative charge cut-off for plotting is −75; refer to Fig. S1c for the full plot). The horizontal dashed line divides the data into positively and negatively charged proteins. For panels C to E, averages of proteins identified in the mass spectrometric analysis from three independent experiments (light gray circles); ribosomal-binding proteins (dark gray circles); AlgP(red circles); polyP kinases(blue circles); polyhydroxyalkanoate granule-associated protein PhaF(green circles); and CHAD proteins (orange circles).

We developed a protocol for enrichment of polyP granules and analysis of granule-associated proteins. We had two objectives when choosing a culture condition for granule isolation: (i) a condition in which polyP granules are evenly spaced, because we are interested in identifying protein factors that may contribute to spatial positioning of granules; and (ii) a condition where polyP granules are abundant, and the presence of polyhydroxyalkanoate (PHA) granules is minimized, to avoid cross-contamination by proteins associated with PHA granules. Three hours of nitrogen starvation in minimal medium satisfied both of these criteria, as shown in our previous work (10). Briefly, we fractionated lysates of WT (wildtype) and a ΔpolyP strain (a quadruple knockout of *ppk1*, *ppk2A*, *ppk2B*, and *ppk2C*) by ultracentrifugation in a self-forming Percoll gradient (19) (Fig. 1A). We observed a brownish-yellow pellet at the bottom of the WT gradient, but not the ΔpolyP gradient (Fig. 1B). We extracted proteins from the pellet and whole lysate of WT and subjected them to label-free proteomic analysis (see Materials and Methods for details).

10.1128/mBio.02463-21.1 FIG S1 Extended proteomic panels of proteome from the three biological experiments shown in Fig. 1. (A) Schematic of polyP granule enrichment protocol. (B) Average absolute mass fraction of proteins in the “pellet” and “lysate,” obtained from spectral counting shown in parts per million, as in Fig. 1C in the main text, on a linear scale representing the complete and unfiltered proteomic data. (C) Charge of proteins identified in the proteomics analysis plotted against the fold enrichment over the complete charge range (Fig. 1E in comparison shows the data for a negative charge cut-off of −75). Figure labels and cut-offs are same as in Fig. 1. (D) Absolute mass fraction of proteins in the “pellet” and “lysate,” obtained from spectral counting shown in parts per million. Data are shown for a representative experiment. (E) Enrichment of proteins in the pellet plotted against the abundance of proteins in the pellet for a representative experiment. (F) Charge of proteins identified in a representative proteomics experiment plotted against the calculated fold enrichment (negative charge cut-off for plotting: −75). Figure labels and cut-offs are same as in Fig. 1. Download FIG S1, TIF file, 1.9 MB. Copyright © 2022 Chawla et al. 2022 Chawla et al. https://creativecommons.org/licenses/by/4.0/ This content is distributed under the terms of the Creative Commons Attribution 4.0 International license.

We identified approximately 1,200 proteins in the pellet fraction. Average protein abundances for the pellet and lysate from three independent experiments are shown in Fig. 1C. As an initial verification for extraction methodology, we sought to confirm enrichment of known polyP associated proteins, including the previously mentioned polyP kinases (Ppk1, Ppk2A, Ppk2B, and Ppk2C), exopolyphosphatase (Ppx), and two CHAD-containing proteins (PA14_13320 and PA14_26940). Ppk1 was more abundant in the lysate than in the pellet and Ppk2B was not detected in our screen. Ppk2C was enriched and highly abundant in the pellet fraction. Ppk2A was detected in the pellet fraction in one of three replicates with low abundance. Ppx was present in the lysate fraction only. One of the two CHAD-containing proteins, PA14_26940, was consistently detected in the pellet fraction but the other, PA14_13320, was not. We note that some ribosome-associated proteins were more abundant and enriched in the pellet than lysate. However, we excluded this class of proteins from subsequent analysis, as they are known to easily extract under the salt conditions used for our screen (20).

We hypothesized that our granule proteomic screen would identify two broad but non-exclusive classes of proteins: (i) structural proteins that contribute to polyP granule formation, and (ii) client proteins that use polyP for another function, such as kinases that use polyP for phosphorylation reactions. PolyP has one of the highest negative charge densities of any macromolecule in the cell (21). Hence, we decided to focus initially on the first class, which we reasoned would have two key characteristics: high enrichment and positive charge. A plot of enrichment, defined as the ratio of abundance of protein in pellet to the lysate, versus the abundance is shown in Fig. 1D. Ppk2C was one of the nine highly enriched and abundant protein candidates identified in this step. A plot of net protein charge at pH 7 (obtained from the Pseudomonas Genome Database [22]) versus protein enrichment in the polyP pellet is shown in Fig. 1E, revealing one protein, AlgP, that distinctly stood out due to its net charge of +55. In contrast, PhaF, which is a PHA granule-associated protein with a net positive charge +35, was abundant, but not enriched per our screening criteria. AlgP and PhaF share a highly homologous C-terminal domain enriched in closely-spaced lysine residues (see Discussion), and their abundance therefore may be an underestimate due to the trypsin cleavage treatment for mass spectrometry. Based on these abundance, enrichment, and physiochemical criteria, we decided to focus on AlgP for subsequent analysis.

### AlgP localizes to polyP granules.

**FIG 2:**
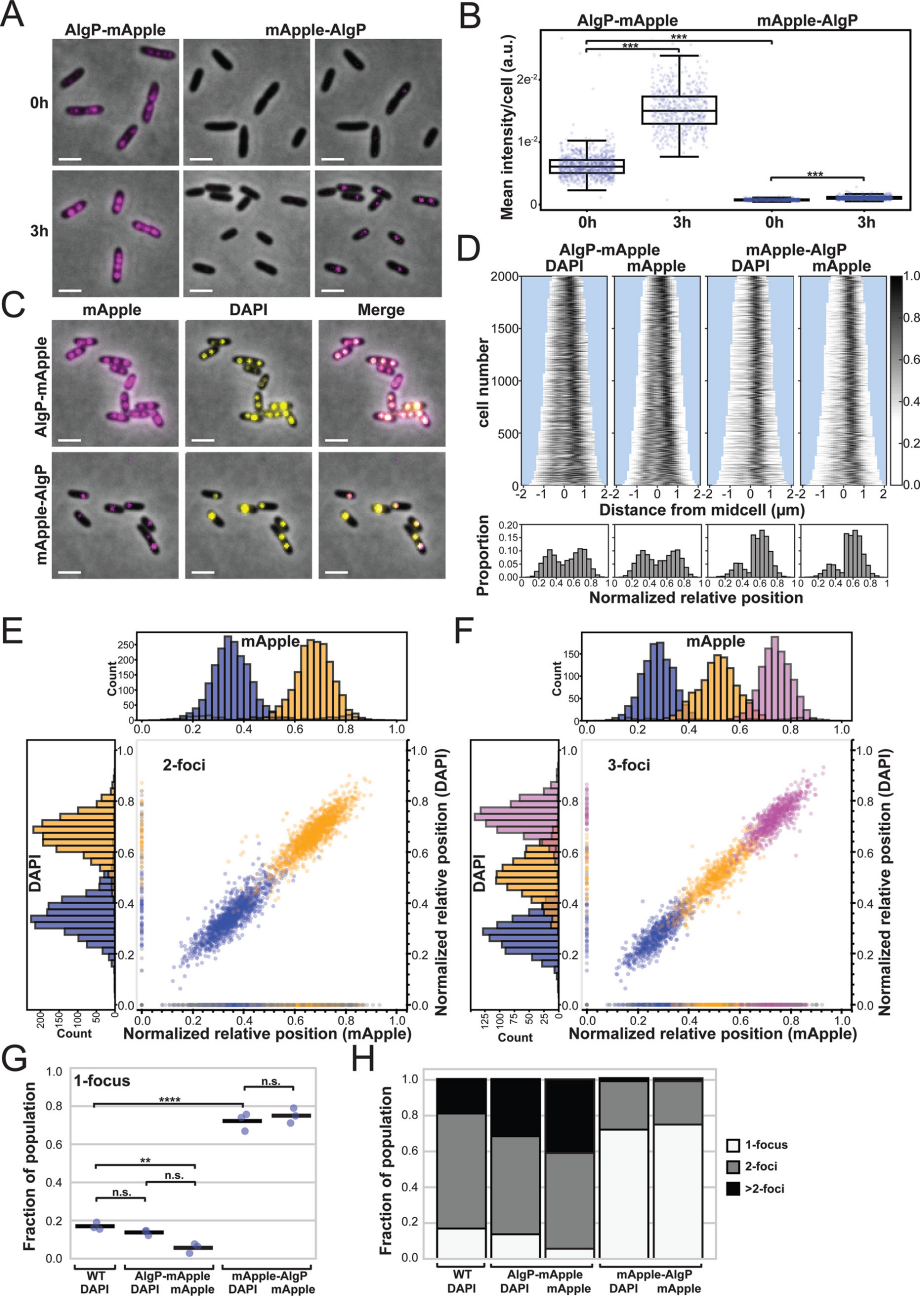
Subcellular localization of AlgP. (A) Representative images of localization of AlgP-mApple and mApple-AlgP translational fusion proteins. The genes encoding the chimeric proteins replaced the endogenous *algP* at its native chromosomal locus. mApple fluorescence displayed in magenta. Left panels, AlgP-mApple in exponential growth (0 h) and after nitrogen starvation (3 h). Middle panels, mApple-AlgP displayed using the same dynamic range as for AlgP-mApple. Right panels, the same mApple-AlgP images, shown with a narrower dynamic range necessary to see puncta. (B) Quantification of the average fluorescence intensity per cell (total intensity divided by the area of the cell) for AlgP-mApple and mApple-AlgP in growing (0 h) and nitrogen-starved (3 h) cells. A Kruskal-Wallis test followed by a Dunn-Bonferroni *post hoc* pairwise test was used to calculate statistical significance, with asterisks showing significant differences as follows: *** indicates *P* < 1e^−5^ (C) Localization of AlgP-mApple and mApple-AlgP relative to DAPI-stained polyP granules. mApple fluorescence is displayed in magenta, DAPI fluorescence is displayed in yellow. Note that while the acquisition settings on the microscope and camera were the same, a different dynamic range was used to display the mApple channel for the AlgP-mApple and mApple-AlgP constructs because of differences in brightness between the constructs. The minimum and maximum pixel intensities displayed in the left and middle panels of (A) for AlgP-mApple and mApple-AlgP are the same (500 a.u. minimum to 3000 a.u. maximum for these 16-bit images), but we show the mApple-AlgP image again in the right panel of (A) with a lower maximum intensity (500 a.u. minimum to 750 a.u. maximum for these 16-bit images). (D) Top: Demographs displaying the fluorescence intensity of DAPI and mApple on the long axis of the cell in 3h nitrogen-starved cells. Demographs display 2,000 cells. Individual cells are flipped such that the brightest segment of the cell appears on the right side of the demograph. Bottom: Histograms display the normalized relative position of fluorescent foci on the long axis of the cell. For individual cells with a single focus, or multiple foci localized to only one half of the cell, the cell is flipped such that all foci for that individual cell appear on the right side of the histogram. (E) Spatial organization of 2-foci cells. Histograms display the normalized relative position on the long axis of the cell of the first foci in blue and second foci in orange. The horizontal histogram (top) represents mApple foci, and the vertical (left) histogram represents DAPI-labeled foci. Dots in the scatterplot show the correlation in spatial position of mApple and DAPI foci. Gray dots represent foci found in one channel but not in the other, these are also displayed as gray in the histograms. Refer to Supplemental Information Methods for a detailed description of how the relative position of spots in the two channels was correlated. (F) Spatial organization of 3-foci cells. As in (E), but the third foci is depicted in magenta. (G) Quantification of the fraction of 1-foci cells in the population in both the DAPI and mApple channel. Note that 0-puncta cells are not included in these calculations, because it is impossible to distinguish between cells that have no granules, and those in which DAPI did not efficiently label the cell. The fraction of 1-foci cells therefore represents the ratio of 1-foci cells to all cells with 1 or more foci. Each point represents an independent experimental replicate performed on different days. Dots represent results from independent experiments on different days, bar shows mean. One-way ANOVA followed by Tukey HSD multiple comparison test was used to calculate statistical significance, with asterisks showing significant differences as follows: **, *P* < 0.01; ****, *P* < 0.0001. (H) Fraction of 1, 2, and >2 foci cells in the population, as in (G). Scale bar, 2 μm.

We used fluorescence microscopy to assess whether AlgP is enriched in polyP granules, as our proteomic analysis suggests. We replaced the endogenous copy of AlgP in the genome with N- or C-terminally tagged fluorescent chimeras of AlgP with mApple. AlgP-mApple forms small irregular puncta and patches in exponentially growing cells (Fig. 2A, top left). After 3h of nitrogen starvation, AlgP-mApple forms discrete evenly spaced puncta on the long axis of the cell (Fig. 2A, bottom left). The mean fluorescence per cell of AlgP-mApple revealed that AlgP is expressed during exponential growth and increases in abundance during starvation (Fig. 2B).

We then used DAPI (4′,6-diamidino-2-phenylindole) staining to compare the localization of these puncta with polyP granules, and qualitatively observe clear co-localization (Fig. 2C, top panels). DAPI undergoes a 90-nm emission shift when bound to polyP relative to DNA, which can be visualized with a custom filter cube (see Supplemental Information Methods). For the AlgP-mApple chimera, demographs of the normalized fluorescence intensity on the long axis of the cell for both DAPI and AlgP-mApple revealed a similar dual banding pattern between the two fluorescent channels (Fig. 2D, top panels). Histograms of the normalized relative position of foci on the long axis of the cell were consistent with this banding pattern, revealing two predominant peaks (Fig. 2D, bottom panels). We compared the number and positioning of DAPI-labeled polyP granules in WT versus AlgP-mApple cells, for all multiple-foci-containing cells, to determine if the chimeric fusion interferes with granule biogenesis. To determine whether the even spacing granule phenotype we have previously observed is preserved in AlgP-mApple-labeled cells, and to quantify co-localization of DAPI and AlgP-mApple labeled foci, we examined the normalized relative position of foci on the long axis of the cell in both channels, for both 2-foci and 3-foci cells (Fig. 2E and F). We observed even spacing in both channels, and the position of individual foci in the two channels was positively correlated in the scatterplot. We also compared the number of foci per cell in cells expressing AlgP-mApple with that of WT, for all foci-containing cells (Fig. 2G and H). We observed a similar fraction of cells with 1 DAPI-labeled focus in WT and AlgP-mApple strains (Fig. 2H), but slightly more cells with 2 DAPI-labeled foci in WT than in the AlgP-mApple strain, and slightly fewer cells with >2 DAPI-labeled foci in WT than in the AlgP-mApple strain (Fig. 2G, Fig. S3A). For the AlgP-mApple strain, the fraction of cells with 1, 2, and >2 foci was similar in the DAPI and mApple channels (Fig. S3A, summarized in Table S3A).

10.1128/mBio.02463-21.3 FIG S3 Effect of AlgP and C-terminus of AlgP on localization of polyP granules during nitrogen starvation and complementation analysis. (A) Quantification of the fraction of cells in the population with 1, 2, and >2 DAPI and mApple-labeled foci per cell. Each point represents an independent experimental replicate performed on different days, the bar indicates the mean. (B) Representative images of DAPI-stained cells for complementation analysis. (C) Quantification of the fraction of cells in the population with 1, 2, and >2 DAPI-labeled foci per cell for the complementation analysis. One-way ANOVA followed by Tukey HSD multiple comparison test was used to calculate statistical significance, with asterisks showing significant differences as follows: *, *P* < 0.05; **, *P* < 0.01; ***, *P* < 0.001; ****, *P* < 0.0001. Scale bar, 2 μm. Download FIG S3, TIF file, 1.1 MB. Copyright © 2022 Chawla et al. 2022 Chawla et al. https://creativecommons.org/licenses/by/4.0/ This content is distributed under the terms of the Creative Commons Attribution 4.0 International license.

10.1128/mBio.02463-21.6 TABLE S1 Summary of proteomics data. The fractional abundance of proteins found in the “pellet” and “lysate” fractions from three independent experiments and their averages are provided in a tabular format. Download Table S1, TXT file, 0.3 MB. Copyright © 2022 Chawla et al. 2022 Chawla et al. https://creativecommons.org/licenses/by/4.0/ This content is distributed under the terms of the Creative Commons Attribution 4.0 International license.

10.1128/mBio.02463-21.7 TABLE S2 (a) Summary of highly abundant and enriched proteins identified in the pellet. The table summarizes the proteins that were both highly abundant in the pellet and highly enriched in the “pellet” fraction. (Abundance cut-off, 5,000 ppm, Enrichment cut-off, 8). These are the proteins found in the upper right quadrant, Fig. 1D. (b) Summary of highly enriched proteins and positively charged proteins identified in granules. The table lists the proteins that were both highly abundant in the pellet and highly positively charged. (The enrichment cut-off was 8-fold, the charge cut-off was +5). This table includes the proteins plotted in the upper right quadrant of Fig. 1E that have a charge greater than +5. Download Table S2, DOCX file, 0.03 MB. Copyright © 2022 Chawla et al. 2022 Chawla et al. https://creativecommons.org/licenses/by/4.0/ This content is distributed under the terms of the Creative Commons Attribution 4.0 International license.

10.1128/mBio.02463-21.8 TABLE S3 (a) Fluorescence foci summary. Quantification of percentages of 1, 2, and >2-foci cells in DAPI and mApple channels. Values represent average and standard deviation of between three to six independent experiments performed on different days. (b) Transmission electron microscopy summary data. Average and standard deviation values calculated from the total population of cells as depicted in Fig. 4. (c) Cell cycle exit. Quantification of percentage of cells with >1 origin per cell and >0 DNA replication forks per cell. Values represent average and standard deviation of three independent experiments performed on different days, as shown in Fig. 6. Download Table S3, DOCX file, 0.02 MB. Copyright © 2022 Chawla et al. 2022 Chawla et al. https://creativecommons.org/licenses/by/4.0/ This content is distributed under the terms of the Creative Commons Attribution 4.0 International license.

The mApple-AlgP chimera exhibited a different behavior. The fluorescence intensity of this chimera was significantly lower both in exponential growth and during starvation, suggesting that the N-terminal tag may interfere with expression, folding, or stability of the full-length protein (Fig. 2B). The localization of mApple-AlgP was different from AlgP-mApple: we still observed puncta, but significantly fewer than we observed with the C-terminal tag (Fig. 2A, right panels, note that we’ve displayed this image using different dynamic ranges, in the middle panel the dynamic range is identical to that used for the AlgP-mApple construct, but on the right, a narrower dynamic range was required in order to see the much dimmer mApple-AlgP foci). The mApple-AlgP protein co-localize with granules, but granule number and organization are significantly perturbed, with significantly more cells displaying a single DAPI-labeled focus than either the WT or the AlgP-mApple strains (Fig. 2C, bottom panels, Fig. 2D, right panels, Fig. 2G and H, and Fig. S3A). Together these observations suggested that AlgP might play a role in the subcellular positioning of polyP granules.

### AlgP affects granule maturation and spatial organization.

**FIG 3:**
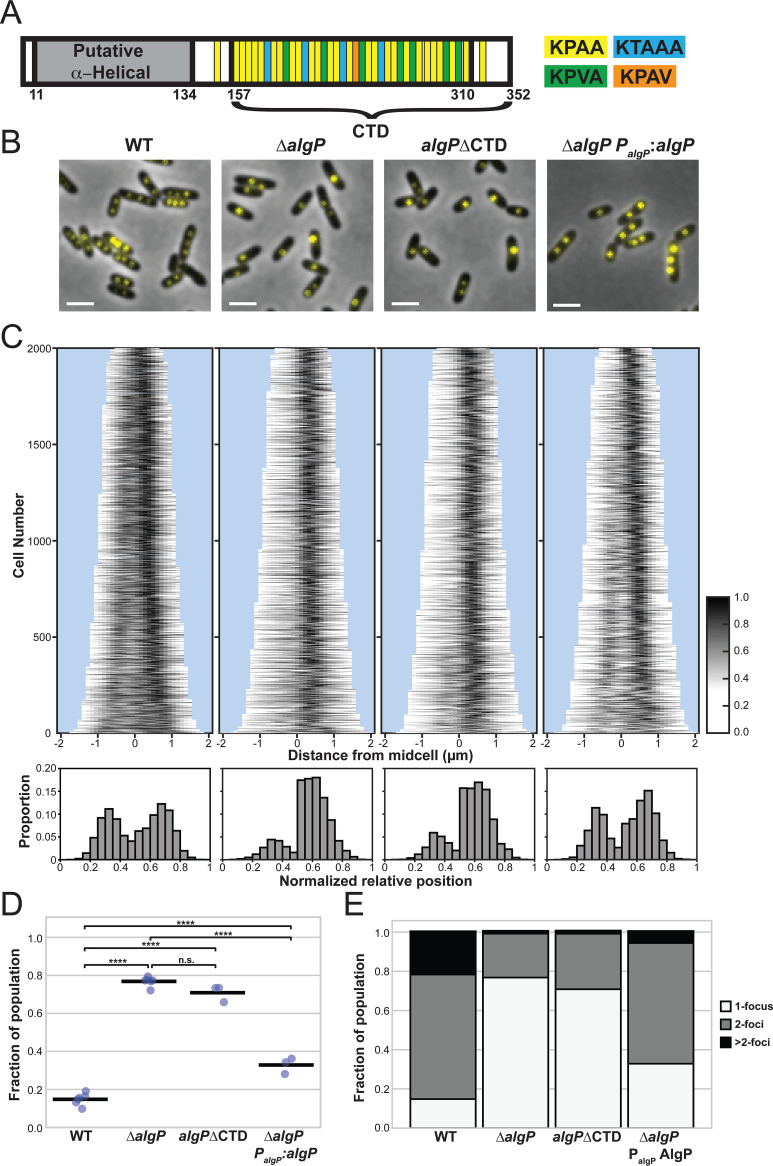
Effect of AlgP and C-terminus of AlgP on localization of polyP granules during nitrogen starvation. (A) AlgP protein domain organization. AlgP contains an N-terminal 124aa putative α-helical domain. The C-terminus contains a 195aa domain predicted to be intrinsically disordered, with a 154 region of tandem repeats of KPAA. There are 25 perfect KPAA repeats interspersed with variants, including 7 KPVA, 4 KTAAA, one KPAV, and two alanine spacers. Two KPAA repeats that fall outside of this 154aa contiguous region. (B) Representative images of DAPI-stained cells. Note that first three panels are from the same day and use the same dynamic range for display, but the fourth panel was from a different day and is displayed with a different dynamic range. For images of the control strains taken on the same day, with the same dynamic range as the complementation strain, please see Fig. S3B. (C) Top: Demographs displaying the fluorescence intensity of DAPI on the long axis of the cell in 3h nitrogen-starved cells. Demographs display 2,000 cells. Individual cells are flipped such that the brightest segment of the cell appears on the right side of the demograph. Bottom: Histograms display the normalized relative position of fluorescent foci on the long axis of the cell. For individual cells with a single focus, or multiple foci localized to only one half of the cell, the cell is flipped such that all foci for that individual cell appear on the right side of the histogram. (D) Quantification of the fraction of DAPI-labeled cells in the population with one focus per cell. Each point represents an independent experimental replicate performed on different days. Note that 0-puncta cells are not included in these calculations, because it is impossible to distinguish between cells that have no granules, and those where DAPI did not efficiently label the cell. The fraction of 1-foci cells therefore represents the ratio of 1-foci cells to all cells with 1 or more foci. Dots represent results from independent experiments/days, the bar indicates the mean. One-way ANOVA followed by Tukey HSD multiple comparison test was used to calculate statistical significance, with asterisks showing significant differences as follows: ****, *P* < 0.0001. (E) Fraction of 1, 2, and >2 foci cells in the population, as in (D). Scale bar, 2 μm.

**FIG 4:**
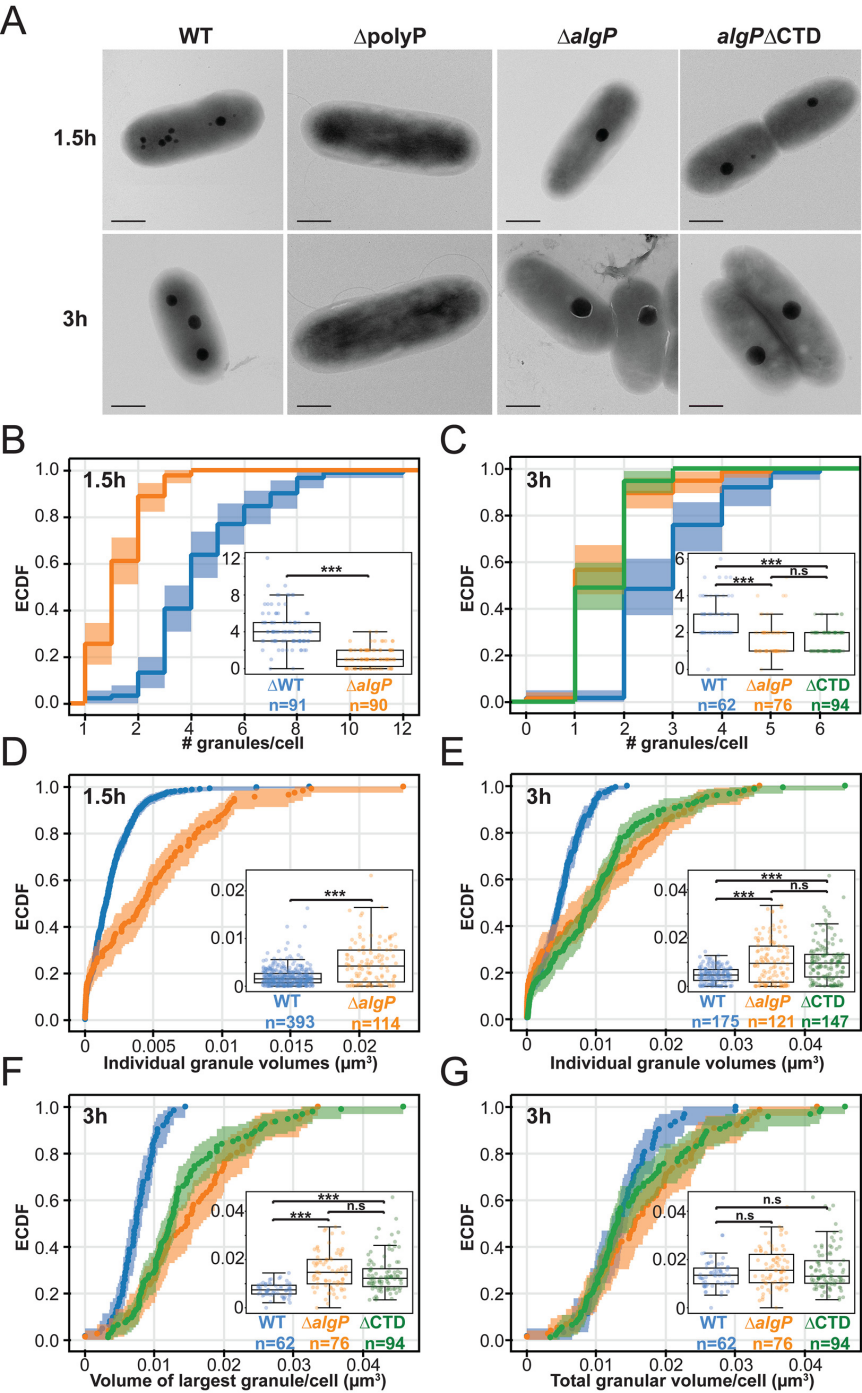
Effect of AlgP and C-terminus of AlgP on number and size of polyP granules during nitrogen starvation. For the empirical cumulative distribution functions (ECDFs), the shading represents confidence intervals for the ECDF acquired by bootstrapping. Inset box/swarm plots show the same data as depicted in the ECDF. (A) Representative TEM images of cells 1.5 h and 3 h after nitrogen starvation. (B) Distribution of the number of granules per cell for WT and *ΔalgP* cells at 1.5 h into nitrogen starvation. (C) Distribution of the number of granules per cell for WT, *ΔalgP*, and *algP*ΔCTD cells at 3 h into nitrogen starvation. (D) Distribution of volumes of all individual granules at 1.5 h into nitrogen starvation for WT and *ΔalgP* cells. (E) Distribution of volumes of all individual granules at 3 h into nitrogen starvation for WT, *ΔalgP*, and *algP*ΔCTD cells. (F) Distribution of volumes of the largest granule per cell after 3 h of nitrogen starvation. (G) Distribution of total granular volume per cell (sum of volumes of all granules within a cell) after 3 h of nitrogen starvation. For panels C, and E to G, a Kruskal-Wallis test followed by a Dunn-Bonferroni *post hoc* pairwise test was used to calculate statistical significance, with asterisks showing significant differences as follows: *** indicates *P* < 1e^−5^. For panels B and D, a Mann-Whitney test was used to calculate statistical significance, with asterisks showing significant differences as follows: ***, *P* < 1e^−5^. Scale bar, 0.5 μm.

AlgP is a 352 amino acid (aa) protein with a 124aa N-terminal domain predicted to be α-helical (Alpha Fold P15276; prediction based on the NTD of P. aeruginosa strain PAO1 AlgP, which is identical to the NTD of AlgP in strain PA14), and a 195aa predicted intrinsically disordered C-terminal domain containing a 154aa region of tandem repeats of KPAA (23, 24). There are 25 perfect KPAA repeats interspersed with variants, including seven KPVA, four KTAAA, one KPAV, and two alanine spacers. Two KPAA repeats fall outside of this 154aa contiguous region (Fig. 3A and Fig. S2). To determine if AlgP is required for polyP granule formation and/or organization, we constructed a clean deletion (*ΔalgP*). Granule number and spatial organization is perturbed in *ΔalgP* cells (Fig. 3B to E, Fig. S3B to C). We performed complementation analysis by inserting a copy of *algP* under its native promoter at the *att*Tn7 locus, and observed partial rescue of granule number and organization (Fig. 3B, E, Fig. SB to SC). Given the limits of light microscopy to detect small granules and to accurately measure granule size, we used transmission electron microscopy (TEM) as an orthogonal method to characterize the granule phenotype in *ΔalgP* cells early in starvation (1.5 h) and at 3 h. We observed fewer, larger granules for *ΔalgP* cells at both time points (Fig. 4A to E). Indeed, when we look at the volume of the largest granule per cell at 3h, this trend toward larger granules becomes very apparent (Fig. 4F). While granules were larger in *ΔalgP* cells than WT cells at 1.5 h, we saw a larger subpopulation of cells without granules at this time point in *ΔalgP* cells (26% of *ΔalgP* cells versus 2% of WT cells). While *ΔalgP* cells have fewer granules, these granules are actually larger, and the loss of AlgP does not appear to lead to a net decrease in the total volume of polyP granules per cell at the 3 h time point (Fig. 4G).

10.1128/mBio.02463-21.2 FIG S2 AlgP protein sequence. (A) The boxed region highlights the 154 residue contiguous repeat domain in the C-terminal domain. Highlighted are 25 perfect KPAA repeats (yellow), interspersed with variants, including 7 KPVA (green), 4 KTAAA (cyan), one KPAV, and two alanine spacers, and two KPAA repeats (yellow) that fall outside of this 154aa contiguous region. (B) Alignment of AlgP and PhaF. Multiple sequence alignment of representative AlgP-like proteins generated using NCBI COBALT tool are shown in C and D. (C) Graphical overview of the multiple sequence alignment of complete protein from representative bacterial species. Highly conserved and less conserved amino acid positions based on the relative entropy threshold of the residue are highlighted. Only alignment positions with no gaps are colored. Red indicates highly conserved positions and blue indicates lower conservation. (D) Graphical overview of the NTD (amino acids 1 to 132) from panel C. Color coding as indicated for C. Details of the strains used for alignment: Pseudomonas aeruginosa PA 14, Pseudomonas aeruginosa PAO1, Pseudomonas viridiflava, Pseudomonas fluorescens, Pseudomonas putida, Pseudomonas syringae, Pseudomonas stutzeri, Acinetobacter baumannii, Klebsiella pneumoniae, *Priestia aryabhattai*, Streptococcus dysgalactiae subsp. equisimilis, Streptococcus pneumoniae, and *Bacillus sp.* TH86. Download FIG S2, TIF file, 1.1 MB. Copyright © 2022 Chawla et al. 2022 Chawla et al. https://creativecommons.org/licenses/by/4.0/ This content is distributed under the terms of the Creative Commons Attribution 4.0 International license.

We note that for the TEM analysis we observe a subpopulation of cells with many small “satellite” granules. We previously observed these small granules of 59 ± 20 nm nanometers in diameter, or smaller, appear only in a fraction of cells (10). We did not quantify these “satellite” granules in our analysis, but we note the fraction of cells that contain them: in 3 h nitrogen-starved cells, we observe these in 24% of WT cells and 26% of Δ*algP* cells. We show examples of cells with “satellite” granules in Fig. S5A.

10.1128/mBio.02463-21.4 FIG S4 Example of “satellite” granules in cells imaged by TEM. (A) Two examples of 3h nitrogen-starved WT cells displaying small satellite granules. (B) Three examples of 0 h (exponential phase) WT cells. Top and middle cells display nascent granules, but the bottom cell does not. Scale bar, 0.5μm. Download FIG S4, TIF file, 2.5 MB. Copyright © 2022 Chawla et al. 2022 Chawla et al. https://creativecommons.org/licenses/by/4.0/ This content is distributed under the terms of the Creative Commons Attribution 4.0 International license.

10.1128/mBio.02463-21.5 FIG S5 Effect of polyP and AlgP on growth. Optical density (500 nm absorbance) as a function of time for P. aeruginosa cultures in MOPS minimal media. Cells were subcultured from overnight cultures where cells were preconditioned in MOPS minimal media. Download FIG S5, TIF file, 0.3 MB. Copyright © 2022 Chawla et al. 2022 Chawla et al. https://creativecommons.org/licenses/by/4.0/ This content is distributed under the terms of the Creative Commons Attribution 4.0 International license.

### Deletion of the KPAA repeat domain results in loss of granule positioning.

The C-terminal 154aa KPAA repeat domain of AlgP is highly homologous to the C-terminus of PhaF (Fig. S2B) (18). In PhaF, this domain is thought to bind DNA and tether PHA granules to the nucleoid and thus contribute to their even distribution within cells. To determine if the KPAA-repeat domain of AlgP plays a role in granule spacing, we generated an AlgP truncation mutant where we removed the last 195 C-terminal amino acids (residues 157 to 352, including the 154aa repeat region, Fig. 3A, Fig. S2). In this *algPΔ*CTD strain, we observe a similar decrease in granule number per cell as with *ΔalgP* cells (Fig. 3A to C). We also observe this decrease in granule number by TEM (Fig. 4A, right panel and Fig. 4C). We saw a similar increase in granule size in *algPΔ*CTD cells as observed in *ΔalgP* cells (Fig. 4E and F).

### AlgP localization depends on polyP kinases.

**FIG 5:**
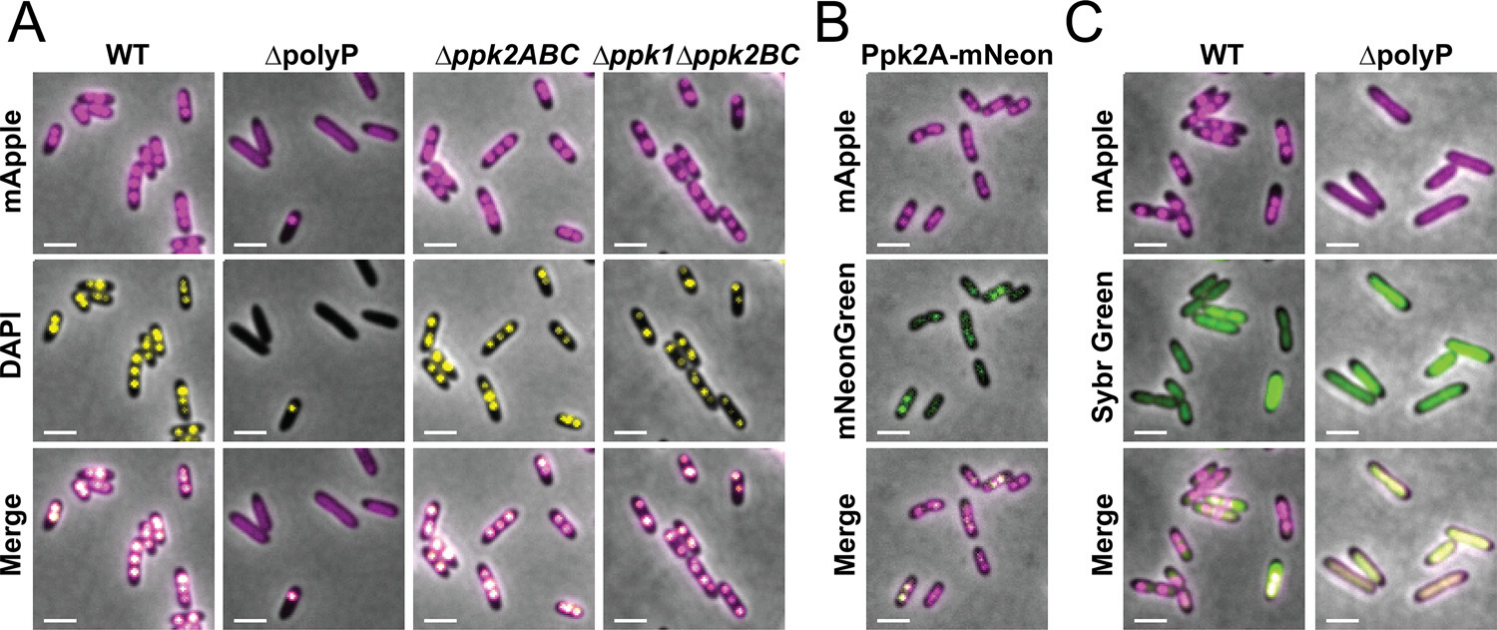
Effects of polyP kinases on AlgP and polyP granule localization after 3 h of nitrogen starvation. (A) Localization of AlgP and DAPI-labeled puncta in the absence of all polyP kinases (ΔpolyP), when only Ppk1 is present (3rd column), and when only Ppk2A is present (4th column). (B) Localization of AlgP-mApple relative to Ppk2A-mNeonGreen. (C) Localization of AlgP relative to the nucleoid in WT and ΔpolyP cells. The nucleoid was visualized using Sybr green labeling (2nd row). Scale bar, 2 μm.

In the absence of starvation, we noticed that AlgP-mApple fluorescence is not diffuse, but already forms puncta (Fig. 2A). These puncta might form due to interactions with the chromosome, a specific polyP kinase, or nascent polyP granules. Indeed at 0 h we observed small putative nascent granules by TEM, though their presence in growing cells is variable (examples shown in Fig. S5B). To determine whether AlgP localization is tied to the polyP polymer itself or instead to the polyP kinases, we expressed our AlgP-mApple fusion in various PA14 strains lacking some or all of the kinases. A mutant lacking all four polyP kinases lacks these puncta, suggesting that the localization depends on polyP and/or specific polyP kinases (Fig. 5A). We note that in *Δ*polyP cells, we occasionally observe cells with a single DAPI stained puncta (<10% of cells), with which AlgP-mApple co-localizes. We do not believe these puncta are polyP granules because of their small size and number per cell, and because we do not see them by TEM (Fig. 4A). We can only speculate about what they might represent, acknowledging that dyes can act non-specifically and DAPI is known to undergo a red emission shift when bound to RNA as well as other biomolecules (25). We next sought to determine whether the AlgP-mApple puncta we observe depend on the polyP polymer, or one of the polyP kinases specifically. We previously showed that either Ppk1 or Ppk2A is sufficient for granule biogenesis (10). In a *Δppk2AΔppk2BΔppk2C* triple mutant, we observe puncta formation, indicating that Ppk1 alone is sufficient for AlgP-mApple puncta formation (Fig. 5A, 3rd column). Similarly, in a *Δppk1Δppk2BΔppk2C* triple mutant, we observe puncta formation, indicating that Ppk2A alone is also sufficient (Fig. 5A, right panels).

We then looked at co-localization of AlgP-mApple with Ppk2A. We previously showed that Ppk2A-mCherry can co-localize with DAPI-labeled polyP granules (10). We qualitatively observe co-localization of Ppk2A-mNeonGreen with AlgP-mApple, although the Ppk2A-mNeonGreen and Ppk2A-mCherry signals are low when these proteins are expressed from the native *ppk2A* promoter and not every DAPI-stained or AlgP-mApple-labeled granule has visible Ppk2A fluorescence (Fig. 5B). We conclude that AlgP-mApple is a brighter and more consistent marker of granules than Ppk2A-mNeonGreen.

Finally, we noticed that the AlgP-mApple fluorescence in ΔpolyP cells was not completely diffuse throughout the cytoplasm (Fig. 5A and C). Instead, we observed apparent co-localization of AlgP-mApple with the nucleoid region of the cell, as visualized by Sybr green staining, suggesting that in ΔpolyP cells, AlgP may bind nonspecifically to DNA (Fig. 5C). Together these data suggest that AlgP-mApple puncta formation depends not on a specific polyP kinase, but rather the presence of small amounts of polyP in growing cells, and that AlgP may bind to the chromosome more broadly in the absence of polyP.

### AlgP is not required for efficient cell cycle exit.

**FIG 6:**
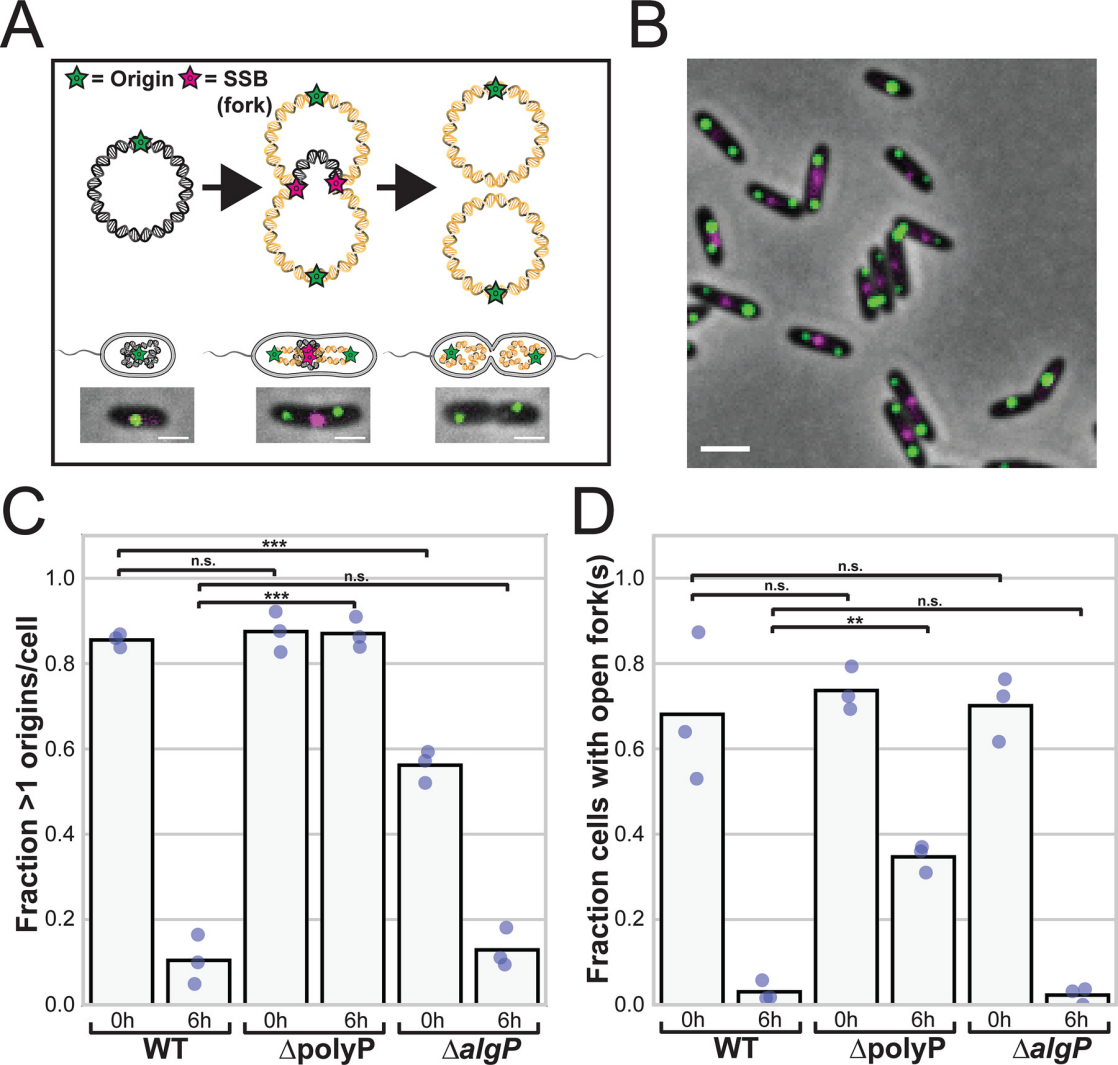
Effects of AlgP on cell cycle exit during nitrogen starvation. (A) Representative image of WT exponentially growing cells with chromosomal origins (green) and open DNA replication forks (red). Origins are labeled using GFP-ParB^pMT1^ and a single copy of its cognate DNA binding site, *parS^pMT1^* DNA sequence at the *attB* locus, 19.5 kb from the origin. Forks are labeled using a fluorescent chimera of single stranded DNA-binding protein (SSB-mCherry). We used a second copy of the SSB promoter to drive both SSB-mCherry and GFP-ParB^pMT1^ expressed from the same operon, and also integrated at the *attB* locus. (B) Fraction of cells with more than one chromosomal origin per cell in growing (0 h) or 6 h nitrogen starved (6 h) cells. Each point represents an independent experimental replicate performed on different days. (C) Fraction of cells with at least one open DNA replication fork in growing (0 h) or 6 h nitrogen-starved cells. Each point represents an independent experimental replicate performed on different days. In (B) and (C), dots represent results from independent experiments/days, and the bar indicates the mean. One-way ANOVA followed by Tukey HSD multiple comparison test was used to calculate statistical significance, with asterisks showing significant differences as follows: **, *P* < 0.01; ***, *P* < 0.0001. Scale bar, 2 μm.

We previously showed that polyP is required for efficient cell cycle exit in response to nitrogen starvation (10). We also previously showed that transient even spacing of granules on the long axis of the cell coincides with the period of cell cycle exit, between 3 h and 6 h into nitrogen starvation (10). We therefore wondered whether AlgP, which is required for granule organization, might also affect efficient cell cycle exit. To characterize the effect of AlgP on cell cycle exit, we used fluorescent markers to label the chromosomal origin of replication and open DNA replication forks, as we have done previously (10). Briefly, we labeled open DNA replication forks with a fluorescent chimera of single stranded DNA binding protein (SSB-mCherry), and we used the heterologous chimeric chromosome partitioning protein GFP-ParB^pMT1^ and its cognate DNA binding sequence *parS*^pMT1^ to label the chromosome near origins (the *parS*^pMT1^ DNA sequence is integrated at the *attB* locus, which is 19.5 kb from the origin). In exponential growth, most WT and ΔpolyP cells are copying their chromosomes, with more than one origin/cell and an open DNA replication fork (Fig. 6B and C, summarized in Table S3c). While we observe a similar fraction of cells with open forks in exponentially growing *ΔalgP* cells, the fraction of cells with more than one origin per cell is significantly lower. However, the bulk growth rates of the WT and *ΔalgP* are similar (Fig. S5), suggesting that the difference we observe with the fluorescent tags does not reflect an underlying replication defect. At 6 h after shifting exponentially growing cells to nitrogen-limited MOPS minimal media, 10±6% of WT and 13±5% of *ΔalgP* cells have more than one origin per cell, and 3±2% of WT and 2±2% of *ΔalgP* cells have open DNA replication forks (Fig. 6B). In contrast, as we previously have shown, ΔpolyP cells have a defect in efficient exit, as 87±4% still had more than one origin/cell and 35±3% had open forks after 6 h of nitrogen starvation (Fig. 6B and C, summarized in Table S3c). These data suggest that AlgP does not play a role in efficient cell cycle exit during nitrogen starvation.

## DISCUSSION

We and others have observed spatial organization of polyP granules in bacteria, and further hypothesized that polyP granules may have an organizational role (6, 26–29). Our granule enrichment protocol led us to identify and validate the protein AlgP as a bona fide polyP associated protein. We have also shown that AlgP has a structural role in granule formation: cells lacking AlgP or the C-terminal domain of AlgP lack evenly spaced granules. How AlgP promotes even spacing of granules remains to be determined, but the amino acid sequence of its C-terminus, along with previous studies of AlgP and related proteins, provide exciting clues.

### Proteomic screen: Proof of principle and next steps.

PolyP has been implicated in diverse roles in bacterial cell physiology, but the identity and function of the proteins that interact with this structurally simple biomolecule *in vivo* have remained elusive. Previous studies have reported isolation of a polyP pellet obtained from centrifugation of lysates in other bacteria including *Desulfovirbiro gigas*, Corynebacterium glutamicum, and Ralstonia eutropha (30–32). In *R. eutropha*, these methods led to enrichment of known polyP kinases, confirmed by fluorescence microscopy. To the best of our knowledge, the enrichment and characterization of polyP granule-associated proteins has not been reported in P. aeruginosa.

Our granule enrichment protocol identified AlgP as a polyP granule-associated protein in P. aeruginosa PA14. Here, the abundance and enrichment characteristics of two known classes of polyP-associated proteins (polyP kinases and conserved histidine α-helical domain (CHAD) motif containing proteins) are worth noting. P. aeruginosa has one known polyP phosphatase (Ppx) and two classes of polyP kinases: one Ppk1 and three Ppk2-type kinases (Ppk2A, Ppk2B, and Ppk2C). In our screen Ppk1 and Ppx were consistently depleted in the pellet fraction (Fig. 1C). Although Ppk2A was enriched in the pellet fraction at only low abundance and was detected in only one of three independent experiments, Ppk2A-mNeonGreen co-localizes with polyP granule (Fig. 5C). Hence, it is possible that Ppk2A weakly or transiently associated with polyP granules and that our granule isolation protocol might have disrupted this association. Ppk2B was not detected in our screen but Ppk2C was consistently and significantly enriched and abundant. Differential association of polyP kinases and phosphatases with polyP granule has previously been observed in betaproteobacterium Ralstonia eutropha H16: Ppk1A*_Reu_*, Ppk2C*_Reu_*, Ppk2D*_Reu_*, and Ppk2E*_Reu_* localize with the polyP granule, whereas Ppk1B*_Reu_*, Ppk2A*_Reu_*, Ppk2B*_Reu_*, and Ppx*_Reu_* are not associated with granules(4, 32). Curiously, AlgP has a highly basic isoelectric point (>11). Previously, it has been found that only *R. eutropha* polyP kinases with a basic isoelectric point co-localized with polyP granules. In P. aeruginosa the presence of the Ppks in isolated polyP granule fraction does not seem to correlate perfectly with their isoelectric point: of the polyP kinases with predicted basic isoelectric points, Ppk2A (8.61) and Ppk2C (8.50) both are enriched in the pellet fraction, but Ppk1 (7.72) was not. Of the enzymes with an acidic isoelectric point, Ppx (6.7) is not enriched in the pellet, and Ppk2B (6.8) was not observed in the lysate and may not be expressed under the experimental conditions. Further work is needed to determine if there are features or properties of proteins that reliably predict granule localization.

Previous efforts to isolate polyP granules from *R. eutropha* using density gradient centrifugation methods with cesium chloride, glycerol, and sucrose gradients were reported to be unsuccessful (32). We have used Percoll for our polyP granule isolation protocol. Percoll consists of colloidal silica particles coated with polyvinylpyrrolidone (PVP) and its physical characteristics like low osmolality, low viscosity, non-toxicity, physiological ionic strength, and pH make it an ideal gradient medium for subcellular particle separation (19, 33). These characteristics may have helped preserve both the intactness of the granules and interactions of proteins with polyP granules, and consequently facilitated polyP granule isolation.

Although our list of protein candidates is comparable with the list of proteins previously identified in Ralstonia eutropha by Tumlirsch et al., it is worth noting that only a few proteins (polyP kinases, CHAD domain-containing proteins, etc.) subsequently emerged to co-localize with the granules and the majority of the proteins were identified to be false-positives (32, 34). It is likely that our list also has high false positive rate and future work will seek to determine the co-localization characteristics of other proteins identified and prioritized in our current screen. Bacterial cells can contain a variety of condition-specific large assemblies, such as inclusion bodies and polysomes, that may co-sediment with polyP granules. In support of this possibility of nonspecific nucleation by polyP granules, proteomic analysis of synthetic membraneless condensates in E. coli was reported to be enriched in a large number of cellular proteins (35). PolyP granules could also non-specifically sequester proteins that normally are not part of granules upon cell lysis.

One class of large subcellular bodies of particular interest in this context are Hfq-based assemblies. Hfq has been suggested to form several types of biomolecular condensates which may have distinct functions in E. coli: two types of RNA-based assemblies, Hfq-foci and H-bodies, both involved in RNA processing during nitrogen starvation, and DNA-based assemblies, specifically the formation of heterochromatin (12, 36, 37). In the context of heterochromatin, Hfq has been shown *in vitro* to form phase separated assemblies with DNA and polyP (12). It is unclear if any of these Hfq-based structures exist in P. aeruginosa, and if these structures are spatially and temporally distinct from polyP granules. While present in P. aeruginosa, Hfq was not enriched in our polyP granule fraction isolated under our experimental conditions (early nitrogen starvation), but Hfq and H-bodies are thought to form in E. coli under deep nitrogen starvation, and future work is needed to determine the relationship between polyP and Hfq in P. aeruginosa. The availability of AlgP as a granule marker will facilitate development of improved polyP granule fractionation and isolation protocols.

### Beyond alginate biosynthesis: A role for AlgP during starvation.

AlgP is a widely conserved protein in the Pseudomonas genus in proteobacteria and present in diverse bacterial species outside of the *Pseudomonadaceae* family (see Fig. S2C, D and Text S1). This protein was previously identified as a transcription factor thought to promote synthesis of alginate, an extracellular polysaccharide that contributes to biofilm production and when overproduced results in a “mucoid” phenotype in P. aeruginosa chronic infections which correlates with a poor prognosis (15, 38, 39). The *algP* gene is adjacent to *algR* and *algQ*, known regulators of alginate biosynthesis. Recently, the putative role of AlgP in alginate biosynthesis in P. aeruginosa has been called into question with the observation that deletion of *algP* in two different mucoid strains of P. aeruginosa, did not result in a nonmucoid phenotype. In the *ΔalgP* mutant, alginate production was not attenuated and the alginate biosynthetic operon was not affected (17). Our discovery that AlgP affects polyP granule localization demonstrates that this protein remains important for starvation physiology. Although our AlgP mutant does not display a defect in cell cycle exit, there are intriguing clues that it may play a role in modulating gene expression and affect anaerobic survival (17). Indeed, AlgP was identified as highly expressed in the metabolically less active regions of P. aeruginosa biofilms using BONCAT labeling, suggesting that starvation upregulates its expression (40). In addition to localizing to granules, suggesting a direct structural role in granule organization, it is also possible that AlgP modulates granule organization indirectly via its effects on gene expression during starvation. While AlgP may not control alginate biosynthesis, polyP and alginate, two polymers associated with fitness during starvation, may nevertheless share a regulatory link. An older study implicated the transcriptional regulator AlgQ (originally called AlgR2) in polyP biosynthesis (41). An *ΔalgQ* mutant of P. aeruginosa has reduced levels of polyP, which can be complemented either by adding back AlgQ, or one of its putative downstream effectors, nucleotide diphosphate kinase (Ndk). The authors attribute the ability of Ndk to rescue polyP synthesis to its role in modulating nucleotide pools, particularly GTP pools. GTP is required to make the stringent response regulator (p)ppGpp, which was long thought to be required for polyP synthesis. More recently, in both E. coli and P. aeruginosa, (p)ppGpp was found not to be required for polyP synthesis (10, 42). Ndk may still influence polyP biosynthesis via its effect on nucleotide pools (10, 42). Further work is necessary to explore the possible functional relationship between alginate and polyP, both important biopolymers in starvation and pathogenesis.

10.1128/mBio.02463-21 TEXT S1 Text contains a description of AlgP conservation, S1 figure legends, and S1 Methods. Download Text S1, DOCX file, 0.1 MB. Copyright © 2022 Chawla et al. 2022 Chawla et al. https://creativecommons.org/licenses/by/4.0/ This content is distributed under the terms of the Creative Commons Attribution 4.0 International license.

### The C-terminus of AlgP: Connecting polyP and bacterial chromatin.

TEM and cryo-electron tomography of diverse bacterial species indicate that polyP granules tend to form in the cell’s nucleoid region (6–9). How and why these negatively charged polyanions associate has been unclear, but their co-localization long suggested there may be a functional and/or structural relationship between polyP granules and bacterial chromatin. Recent work in E. coli has shown that polyP can modulate the DNA-binding specificity of the NAP Hfq, driving Hfq to bind AT-rich sequences and promoting transcriptional silencing of these DNA sequences (12). These findings raise the possibility that polyP may play a more general role in chromatin silencing. While AlgP has not previously been implicated in polyP granule biogenesis, the protein has long been thought to associate with the nucleoid. A fragment of the c-terminal domain (CTD) of AlgP binds DNA, and peptides containing KPAA repeats bind DNA *in vitro* (15, 18). Molecular modeling studies suggest that the CTD may form a helix around DNA (18). A second protein in P. aeruginosa, PhaF, contains a highly homologous CTD to AlgP and is also thought in other Pseudomonads to bind DNA (43–45). PhaF has been shown to play a role in the organization of another class of granular structures, PHA granules, which serve as a cellular store of reduced carbon (43, 44). PhaF is thought to tether PHA granules to the chromosome, and in its absence PHA granules clump together (44). The N-terminus of PhaF contains an amphipathic α helix believed to interact with the hydrophobic surface of PHA granules, as well as an oligomerization domain thought to drive tetramerization (43). The N-terminus of AlgP is predicted to be a helical but its structure and function is not yet known. We were surprised to find that the N-terminally-labeled fluorescent chimera (mApple-AlgP) positioning the fluorescent protein adjacent to the NTD disrupted granule organization, but the C-terminal fusion did not affect granule spacing, particularly given the importance of the CTD in granule spacing. The decreased fluorescence we observed in the mApple-AlgP chimera relative to the AlgP-mApple construct suggests that fusing mApple to the NTD may have disrupted expression, folding, or stability of AlgP. The fact that there was no granule spacing defect when we fused mApple to the CTD indicates that any steric effects of mApple on the CTD domain were minimal. This may be due to the flexibility and large number of KPAA repeats in the CTD. Future studies varying the number of KPAA repeats are needed to better understand the role of this domain in granule spacing.

In addition to the sequence homology to PhaF, AlgP has interesting similarities in terms of sequence composition to the intrinsically disordered C-terminal tail of eukaryotic histone H1, also rich in lysine, proline, and alanine, as has been previously noted (14, 15, 17). Unlike the other core histones which are thought to have evolved from archaea, the histone H1 family is thought to be of bacterial origin (46). Histone H1 in eukaryotes plays a role in chromatin condensation, and its CTD interacts with linker DNA flanking the nucleosome (47). We observed that in the absence of polyP kinases, AlgP appears to globally interact with the nucleoid. Further chromatin immunoprecipitation studies are needed to understand its sequence preference *in vivo*. While the oligomeric state of AlgP is not yet known, it is possible that the N-terminus might mediate higher order oligomers that are competent to interact with both polyP and DNA. The recently described role of polyP in mediating heterochromatin assembly with Hfq raises the question of what role AlgP might play in modulating not only polyP granule localization, but nucleoid organization and gene expression more globally.

### The C-terminus of AlgP: Mediator of phase separation?

**FIG 7:**
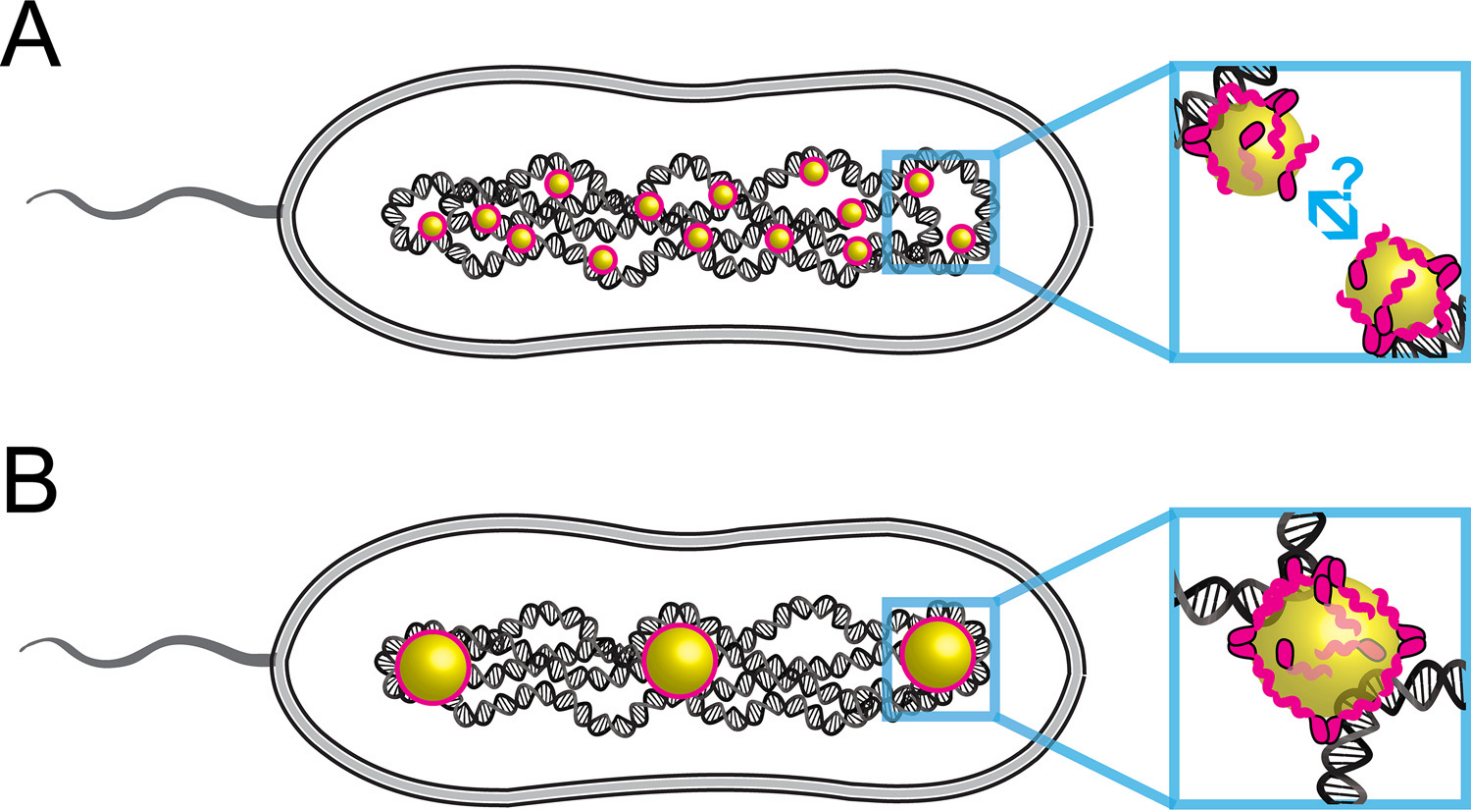
Model for the role of AlgP in granule biogenesis. (A) AlgP tethers nascent polyP granules to the nucleoid, either through specific or nonspecific contacts. Interactions between nascent polyP granules and the nucleoid prevent free diffusion of granules, and lead to some degree of local confinement within a chromosomal “neighborhood.” Such confinement allows granules to fuse only with their nearest neighbors on the timescale of granule maturation (hours). (B) Mature granules, with their larger mass and more extensive interactions with the nucleoid, are more limited in their radius of gyration and do not fuse with other granules, leading to even spacing.

A growing list of phase separated microcompartments have been observed in bacteria and are thought to enable cells to achieve subcellular organization and generate specific microenvironments in the absence of membrane-bound organelles (48, 49). Formation of such compartments is driven by polyanions such as RNA, and intrinsically disordered proteins such as PopZ (50–54). Recently, polyP has been shown to form liquid-like condensates with polycationic peptides and supercharged GFP (+36GFP) (55–57). Additionally, the synthetic +36GFP-polyP condensates were shown to coalesce and exhibit liquid-like properties in Citrobacter freundii *in vivo* (55). PolyP also interacts with the nucleoid associated protein Hfq from E. coli, and together with DNA forms 3-component liquid droplets *in vitro*, and polyP appears to modulate the properties of these Hfq liquid droplets (12). We don’t see a decrease in total granular volume in *ΔalgP* cells, as might be expected if the protein contributed to phase separation of polyP granules. Phase separation of polyP could be driven largely by homotypic (polyP-polyP) interactions mediated by divalent cations such as Mg^2+^, as we and others have previously shown that bacterial polyP granules are indeed enriched in divalent cations (10, 29, 58). But it is also possible that proteins contribute to polyP phase separation via heterotypic (protein-polyP) interactions, whereby positively charged residues interact electrostatically with the negatively charged phosphoryl groups of polyP, as has been observed for other polyanions like RNA (59, 60). AlgP is a candidate for such heterotypic interactions, because of the low complexity, positively charged lysine-rich CTD. If AlgP were contributing to such complex coacervation, one might expect granules to be smaller in the *ΔalgP* mutant, but it is also possible that there is sufficient degeneracy that other players can satisfy this role in the absence of AlgP. Although there is no apparent decrease in granular volume in *ΔalgP* cells, we do see a change in how polyP is distributed between granules, with fewer, larger granules. We speculate that AlgP may normally function to limit granule fusion (Fig. 7). Fusion inhibition might occur by two mechanisms: nonspecific or specific interactions of AlgP with DNA when bound to small/nascent polyP granules could restrict their motion such that they can only fuse with local granules tethered within a given chromatin neighborhood (Fig. 7A). Such restriction to fusing with local neighbors could lead to even spacing. Another non-exclusive model is that AlgP changes the biophysical properties of granules in a manner that inhibits fusion, such as modulating their surface charge. Future time-lapse imaging experiments of polyP granules are needed to address these models. The fusion behavior of polyP granules in the Δa*lgP* mutant in P. aeruginosa differs from the fusion behavior of PHA granules in the Δ*phaF* mutant in another Pseudomonad, Pseudomonas putida. In that species and strain, PHA granules bunch together but do not fuse, likely due to the effect of another protein PhaI (44).

### Functional significance of AlgP in starvation fitness and pathogenesis.

Having discovered that AlgP modulates granule spacing in cells, the next important question is why cells invest in granule spacing at all. Even spacing of another class of bacterial membraneless compartments, carboxysomes, is important for the fitness of daughter cells and mediated by a Brownian-ratchet mechanism (61, 62). While we don’t yet understand the molecular mechanism fully underpinning AlgP-mediated spacing, we now have the means in P. aeruginosa to disrupt polyP granule organization and ask whether even spacing of polyP granules poses either a survival benefit during starvation, or a growth advantage when nutrients become available. The CTD of AlgP has been identified as a hot spot for genetic rearrangements in clinical isolates of P. aeruginosa from cystic fibrosis patients (15, 16). These rearrangements alter KPAA repeat number, suggesting that this domain is under selective pressure during chronic infections, however more research is needed to determine whether there is a connection between these rearrangements and infection outcomes. The consequences of these alterations in AlgP CTD domain length for granule biogenesis, starvation fitness, and pathogenesis remain to be explored. Recently an alkyne derivative of the natural product elegaphenone, which has antibiotic effects against P. aeruginosa, was shown to bind AlgP. If AlgP proves to indeed be important for pathogenesis and elegaphenone treatment mimics the effect of an *algP* mutation on polyP granule localization, AlgP may be worthy of further exploration as a possible drug target (63). As noted previously, AlgP is present in diverse bacterial species, including some medically relevant bacteria. To the best of our knowledge, AlgP is an uncharacterized protein of unknown function in all other species and our current understanding of this protein, including the initial discovery, comes from work in P. aeruginosa. Although we find it tempting to speculate that AlgP might play a role in polyP granule spacing in bacteria other than P. aeruginosa, future work will be needed to determine if this function is more widely conserved.

## MATERIALS AND METHODS

### Media and growth conditions.

For nitrogen starvation experiments, strains were grown overnight at 37°C shaking in MOPS-buffered minimal media (MMM): 40 mM sodium succinate, 22 mM NH_4_Cl, 43 mM NaCl, 2.2 mM KCl, 1.25 mM NaH_2_P0_4_, 1 mM MgSO_4_, 0.1 mM CaCl_2_, 7.5 μM FeCl_2_·4H2O, 0.8 μM CoCl_2_·6H2O, 0.5 μM MnCl_2_·4H2O, 0.5 μM ZnCl_2_, 0.2 μM Na_2_MoO_4_·2H2O, 0.1 μM NiCl_2_·6H2O, 0.1 μM H_3_BO_3_, and 0.01 μM CuCl_2_·2H2O, 50 mM MOPS, pH 7.2. Optical density was determined at 500 nm, rather than 600 nm, to minimize interference of pigments such as phenazines that P. aeruginosa produces during starvation. The 5 mL subcultures at OD500 = 0.0125 to 0.025 were grown in glass test tubes at 37°C shaking at 250 rpm to OD500 = 0.4 to 0.6, then spun down at room temperature for 5 min at 5000xG, and resuspended to OD500 = 0.4 in nitrogen-limited MMM (identical to MMM, but with 1 mM NH_4_Cl instead of 22 mM) in clean glass test tubes and grown at 37°C shaking at 250 rpm. Time 0h = cells collected immediately before being spun down and shifted to nitrogen-limited medium.

### Granule isolation and proteomic analysis.

Refer to the Supplemental Information Methods for a detailed description of cell culturing (cell growth and harvesting) and proteomic analysis (sample preparation, data acquisition, software used, and data availability). Briefly, cells were grown to exponential phase at 37°C in MMM and then shifted to a low-nitrogen MMM for 3 h at 37°C. Cell pellets corresponding to 100 mL of cell culture were flash frozen in liquid nitrogen. Pellet was resuspended and incubated on ice for 15 min in 1 mL of lysis buffer, followed by microtip sonication (Qsonica Q700), and additional nucleic acid digestion with Benzonase and DNase I. Lysate (0.5 mL) was loaded onto 7 mL pre-chilled Percoll gradient solution in ultracentrifuge tubes and spun in a 50TI rotor at 21,300 RPM for 15 min. After removing the gradient solution, a Percoll-encased pellet was resuspended in 1 mL of dilution buffer and centrifuged in a tabletop microcentrifuge at 10,000 × g for 2 min, resuspended, and the spin repeated to remove residual Percoll from the pellet. The pellet was flash frozen in liquid nitrogen for subsequent proteomic analysis. The (g/g) absolute mass fraction of a protein in a given sample was estimated using the spectral counting technique (64). For a protein, its mass fraction abundance was tabulated by dividing the total number its peptide-spectrum matches (PSM) by the total of all 14N PSMs in the sample. Enrichment of a protein in our proteomics screen was defined as the ratio of the abundance of protein in the pellet to the abundance in the lysate. See Supplemental Information Methods for more details.

### Plasmids and strains.

Strains, plasmids, and primers used in this study are listed in Tables S4a to c, respectively. Strain construction was performed as described previously, with details outlined in Supplemental Information Methods (10).

10.1128/mBio.02463-21.9 TABLE S4 (a) Strains. (b) Plasmids. (c) Primers. Download Table S4, DOCX file, 0.02 MB. Copyright © 2022 Chawla et al. 2022 Chawla et al. https://creativecommons.org/licenses/by/4.0/ This content is distributed under the terms of the Creative Commons Attribution 4.0 International license.

### Fluorescence microscopy.

All live cell imaging was acquired with a Nikon Ti2-E inverted microscope. Details of optical setup and acquisition parameters are described in Supplement Information Methods. Cells were imaged at 26°C on 1% agarose pads containing MMM or MMM lacking ammonium chloride for nitrogen starvation experiments. Agarose pads were imaged inside coverslip bottom dishes with plastic lids (Willco Wells, 0.17 mm/#1.5). For DAPI-stained imaging of polyP granules, live cells were stained for 20 min in minimal media with 200 μM DAPI from a 10 mM stock in dimethyl sulfoxide. DAPI staining of live cells for the purpose of polyP granule imaging is not 100% efficient, with some cells not taking up enough dye to label granules (to overcome this limitation of DAPI labeling, we used TEM to quantify the fraction of granule-free cells). But DAPI can be used to characterize the number of granules per cell in cells that take up the DAPI dye, and their organization. For Sybr green staining, cells were stained for 20 min at 1× dye in minimal media before imaging.

### Transmission electron microscopy.

Unfixed cells (3 μL) were spotted onto glow discharged carbon-coated 200 mesh copper grids with pinpointer grid (Ted Pella 01841-F) for 45 s, blotted with Whatman paper, and media salts were washed off grids by spotting the grids with 3 μL of water and rapidly blotting; wash step was repeated 5 times. Samples were analyzed at 120 kV with a Thermo Fisher Talos L120C transmission electron microscope and images were acquired with a CETA 16M CMOS camera.

### Image analysis.

Cell segmentation and spot detection for fluorescence microscopy images was performed using MicrobeTracker (65). Spot detection was performed after rolling-ball background subtraction in Fiji (radius 10 pixels) (66). The SpotFinderZ tool, which fits spots to a Gaussian (see supplemental information for spotFinder parameters) was used, following by manual correction with SpotFinderM. Demographs of fluorescence intensity were constructed using the method and script developed by the Jacobs-Wagner Lab, which generates segments on the long axis of the cell, and then integrates the total fluorescence intensity per segment. It then normalizes the fluorescence of each of these segments to the brightest segment in the cell. We used the “randomNOriented” parameter, which flips cells such that the brightest segment appears on the right side of the demograph (67, 68). For TEM imaging, granule number and diameter per cell were measured manually using Fiji as described previously (10).

### Statistical analyses and data plotting for microscopy.

Statistical analyses was performed using either MATLAB version 2014b or Scipy in Python (69). The statistical tests are indicated in the figure itself or the figure legend. Data were plotted with Bokeh version 2.3.0 and Seaborn version 0.11.1 (70). TEM data was plotted using iqplot version 0.2.1: http://dx.doi.org/10.22002/D1.1614. Fig. 1A and Fig. S1A were created with BioRender.com. Figures were assembled in Adobe Illustrator.

### Data availability.

The mass spectrometry proteomics data have been deposited and will be available upon publication to ProteomeXchange Consortium via the UCSD’s MassIVE repository with the accession codes: MassIVE: MSV000087218 and ProteomeXchange: PXD025444. The raw light and electron microscopy data, plasmid maps, and code are available at zenodo.org: 10.5281/zenodo.6172994 and 10.5281/zenodo.6172996.
