# Supplementary figures and images for: The Histone H1-Like Protein AlgP Facilitates Even Spacing of Polyphosphate Granules in Pseudomonas aeruginosa
Source: mBio. 2022 Apr 18;13(3):e02463-21. doi: 10.1128/mbio.02463-21 (PMC9239181; doi:10.1128/mbio.02463-21)

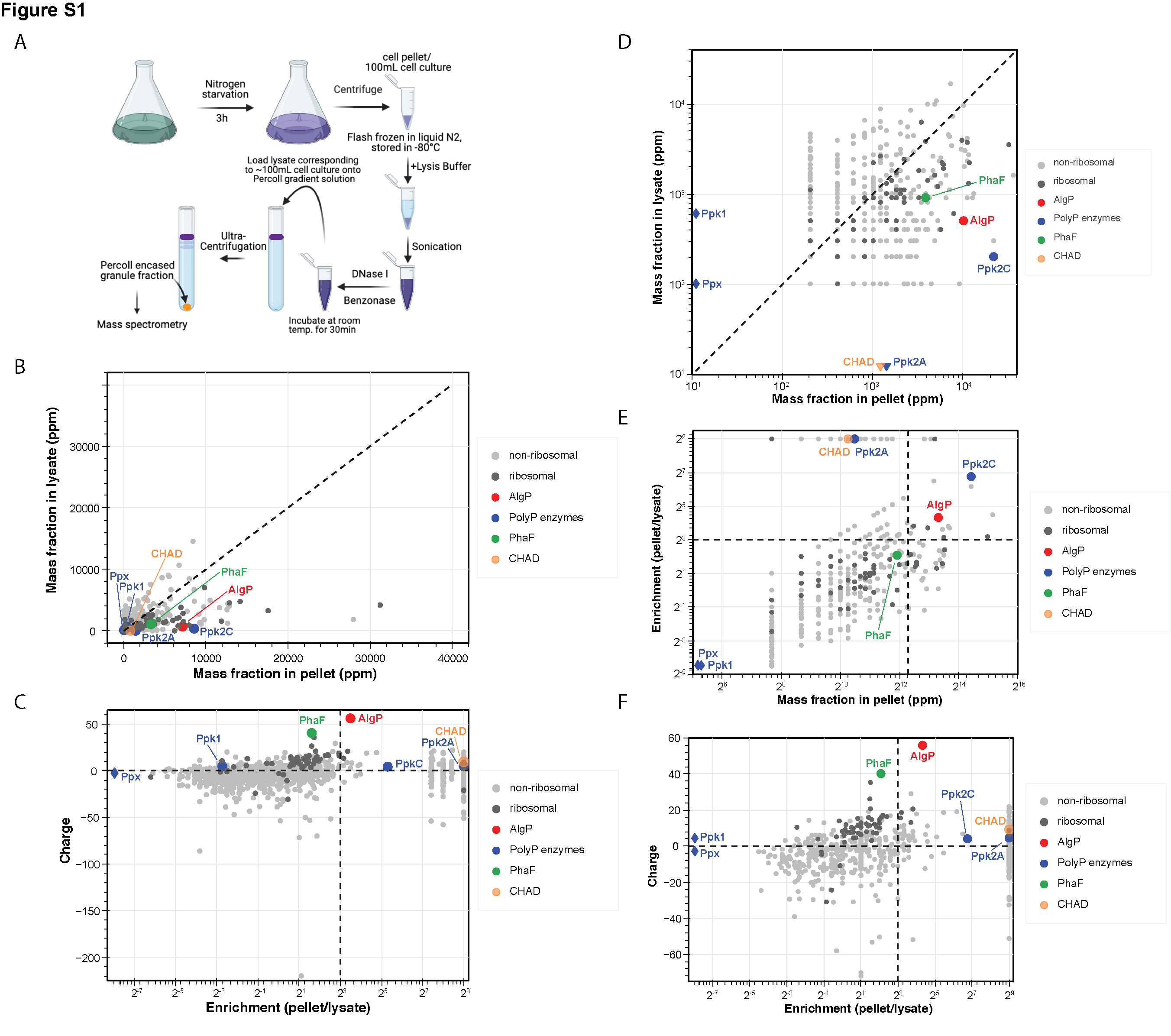

Supplement: FIG S1 [file mbio.02463-21-sf001.tif]

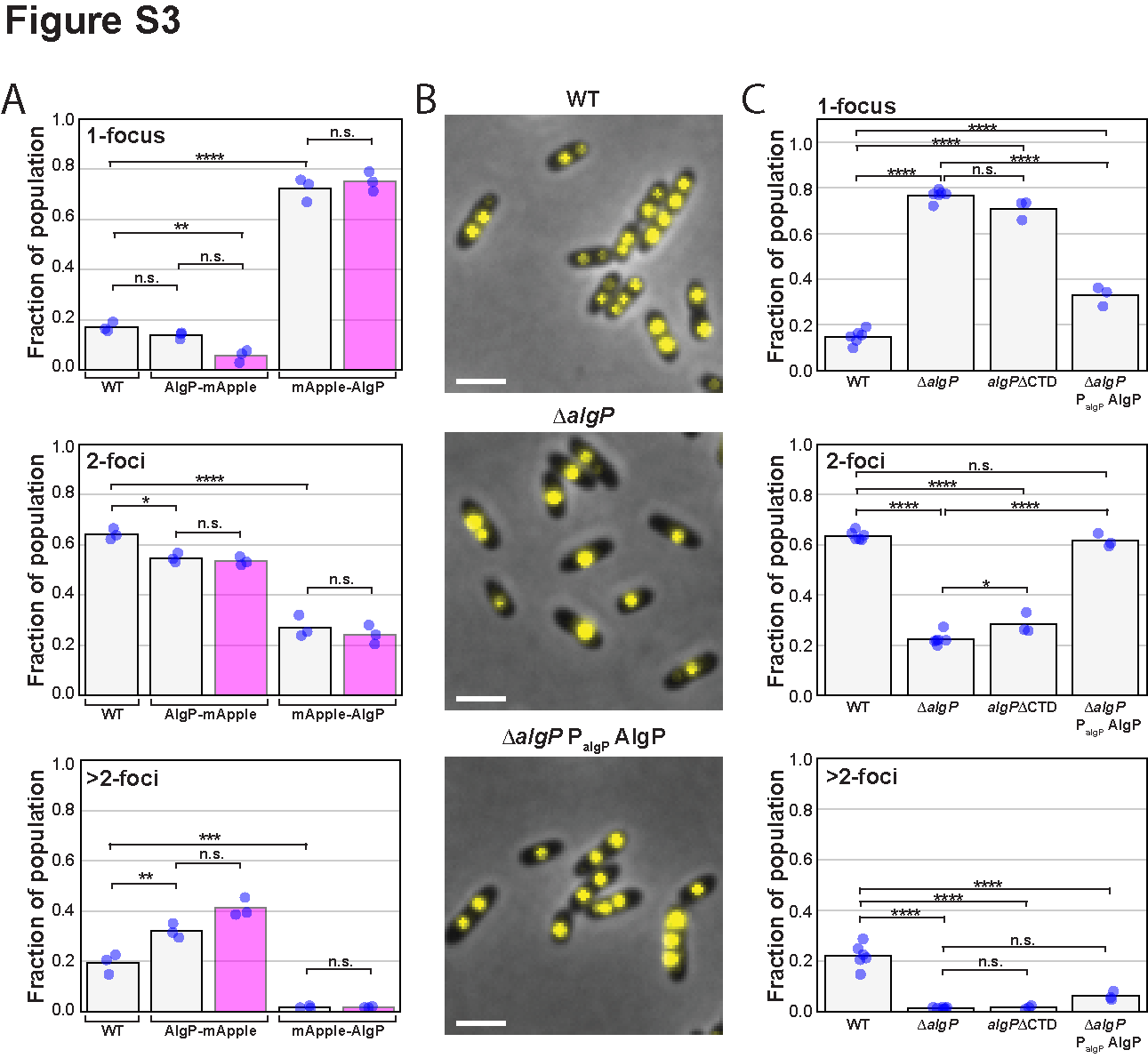

Supplement: FIG S3 [file mbio.02463-21-sf003.tif]

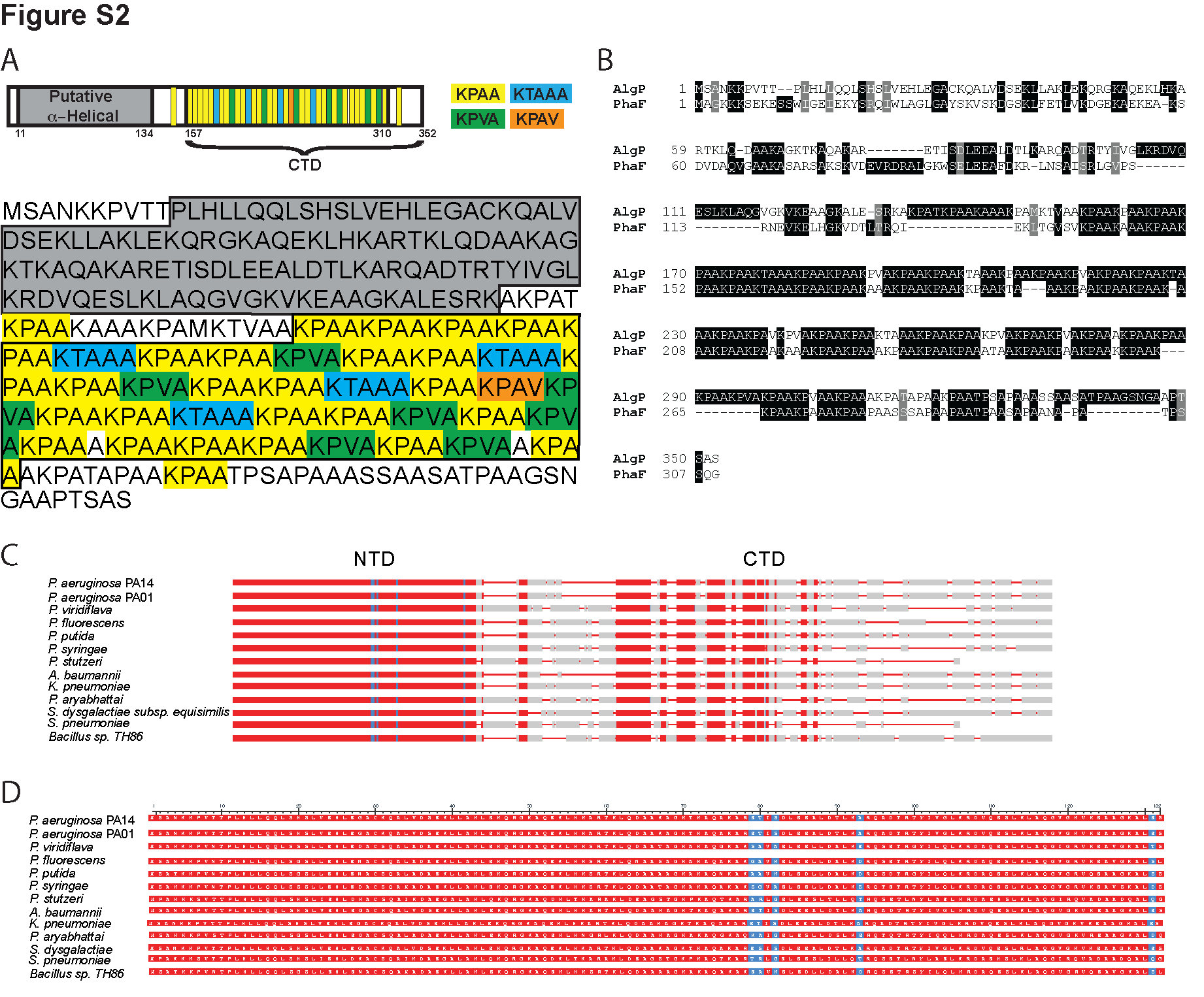

Supplement: FIG S2 [file mbio.02463-21-sf002.tif]

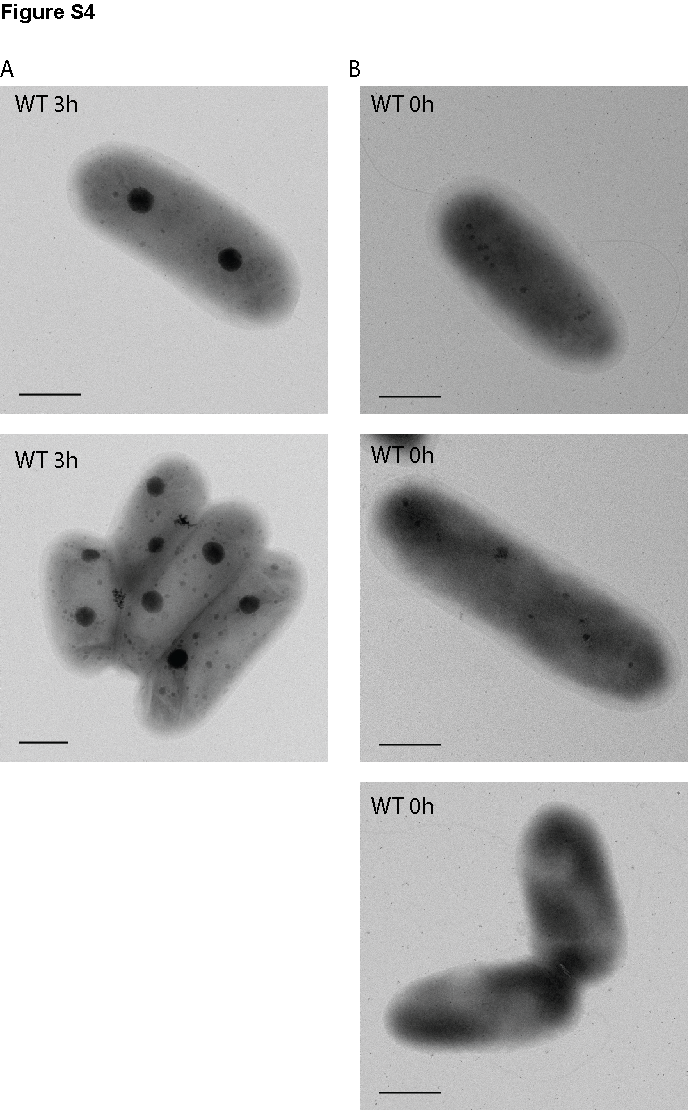

Supplement: FIG S4 [file mbio.02463-21-sf004.tif]

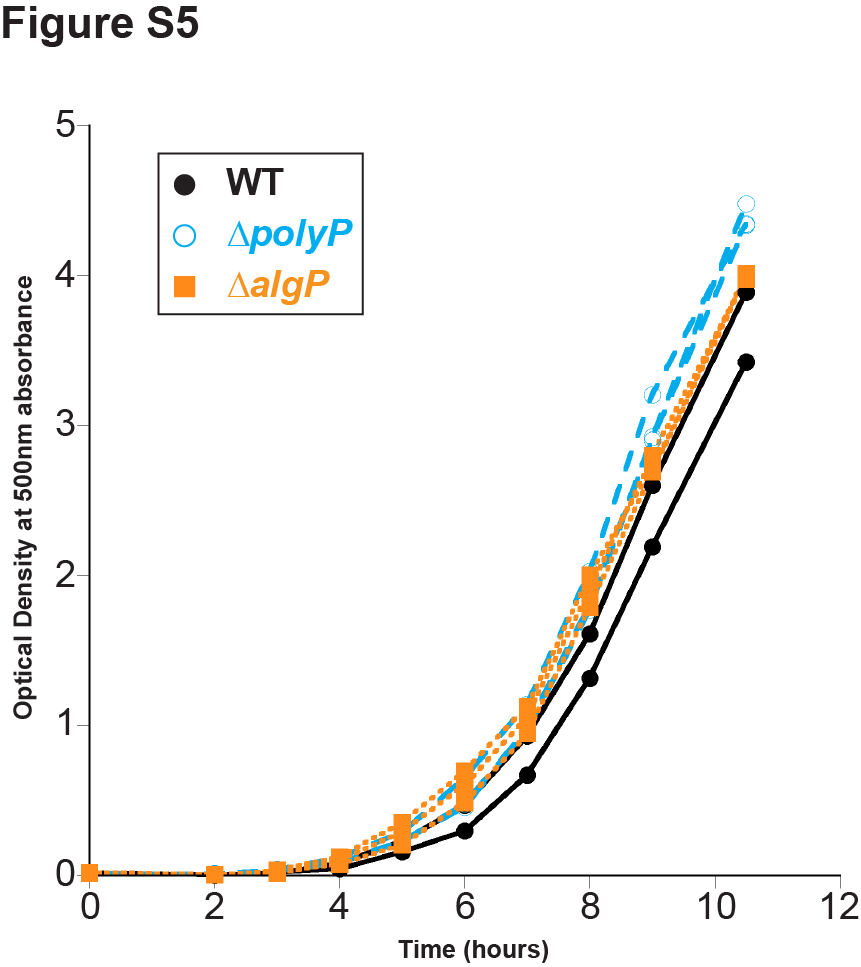

Supplement: FIG S5 [file mbio.02463-21-sf005.tif]
