# Supplementary material for: The Histone H1-Like Protein AlgP Facilitates Even Spacing of Polyphosphate Granules in Pseudomonas aeruginosa
Source: mBio. 2022 Apr 18;13(3):e02463-21. doi: 10.1128/mbio.02463-21 (PMC9239181; doi:10.1128/mbio.02463-21)
Supplement: TABLE S2 [file mbio.02463-21-st002.docx]

**Table S2a: Summary of highly abundant and enriched proteins identified in the pellet**

| Locus tag | Gene | Description | Average Fraction abundance (pellet) | Average Enrichment (Pellet/Lysate) | Estimated Charge  (pH 7) |
| --- | --- | --- | --- | --- | --- |
| PA14_07560 | rpsU | 30S ribosomal protein S21 | 6598.21683 | 8.958031449 | 13.19 |
| PA14_08790 | rpsL | 30S ribosomal protein S12 | 7938.981943 | 10.31125836 | 20.61 |
| PA14_19410 | ppk2C | Polyphosphate kinase | 8551.646369 | 39.5507778 | 4.14 |
| PA14_29590 | Unknown | Putative transcriptional regulator | 9409.912338 | 8.903982375 | 5.47 |
| PA14_49740 | Unknown | Uncharacterized protein | 6244.541078 | 222.9901809 | 6.83 |
| PA14_56070 | mvaT | Transcriptional regulator MvaT, P16 subunit | 27969.53852 | 15.482892 | 6.47 |
| PA14_65200 | rnr | Ribonuclease R (RNase R) (EC 3.1.13.1) | 12620.7254 | 27.19592607 | 19.14 |
| PA14_68660 | rimK | Probable alpha-L-glutamate ligase (EC 6.3.2.-) | 6165.75888 | 512 | 8.83 |
| PA14_69370 | algP | Alginate regulatory protein AlgP | 7151.683224 | 11.27486611 | 55.91 |

**Table S2b: Summary of highly abundant and enriched proteins identified in the pellet**

| Locus tag | Gene | Description | Average Enrichment (Pellet/Lysate) | Estimated Charge (pH 7) |
| --- | --- | --- | --- | --- |
| PA14_69370 | algP | Alginate regulatory protein AlgP | 11.2748661 | 55.91 |
| PA14_12760 | Unknown | Probable ATP-dependent RNA helicase | 350.866132 | 21.87 |
| PA14_19290 | srmB | Putative ATP-dependent RNA helicase | 186.46567 | 21.09 |
| PA14_08790 | rpsL | 30S ribosomal protein S12 | 10.3112584 | 20.61 |
| PA14_65080 | ygiR | UPF0313 protein PA14_65080 | 512 | 19.74 |
| PA14_66210 | waaX | Putative lipopolysaccharide core biosynthesis protein | 256.5296 | 19.35 |
| PA14_65200 | rnr | Ribonuclease R (RNase R) (EC 3.1.13.1) | 27.1959261 | 19.14 |
| PA14_16860 | plsB | Glycerol-3-phosphate acyltransferase (GPAT) (EC 2.3.1.15) | 172.078933 | 19 |
| PA14_15350 | Unknown | Putative integrase | 19.3861906 | 18.67 |
| PA14_25000 | slt | Putative soluble lytic transglycosylase | 256.5296 | 18.29 |
| PA14_66720 | priA | Primosomal protein N' (EC 3.6.4.-) (ATP-dependent helicase PriA) | 512 | 16.62 |
| PA14_64490 | Unknown | Rho_N domain-containing protein | 512 | 16.47 |
| PA14_37820 | Unknown | DUF2235 domain-containing protein | 512 | 14.51 |
| PA14_66190 | Unknown | Uncharacterized protein | 175.256533 | 14.3 |
| PA14_63060 | smpB | SsrA-binding protein (Small protein B) | 512 | 13.85 |
| PA14_53590 | Unknown | Uncharacterized protein | 171.725866 | 13.33 |
| PA14_07560 | rpsU | 30S ribosomal protein S21 | 8.95803145 | 13.19 |
| PA14_69710 | xerC | Tyrosine recombinase XerC | 512 | 13.1 |
| PA14_05970 | Unknown | Uncharacterized protein | 171.019733 | 12.86 |
| PA14_73420 | rnpA | Ribonuclease P protein component (RNase P protein) (RNaseP protein) (EC 3.1.26.5) (Protein C5) | 512 | 12.19 |
| PA14_11080 | cupB3 | Usher CupB3 | 512 | 11.55 |
| PA14_25450 | lolE | Putative lipoprotein releasing system, permease protein | 512 | 11.38 |
| PA14_57340 | murG | UDP-N-acetylglucosamine--N-acetylmuramyl-(pentapeptide) pyrophosphoryl-undecaprenol N-acetylglucosamine transferase (EC 2.4.1.227) (Undecaprenyl-PP-MurNAc-pentapeptide-UDPGlcNAc GlcNAc transferase) | 256 | 11.25 |
| PA14_23410 | orfJ | Putative glycosyl transferase | 180.542645 | 11.22 |
| PA14_24665 | Unknown | THUMP domain-containing protein | 256 | 11.01 |
| PA14_00060 | Unknown | Putative acyltransferase | 512 | 10.86 |
| PA14_08440 | Unknown | Putative short chain alcohol dehydrogenase | 512 | 10.63 |
| PA14_24620 | Unknown | Uncharacterized protein | 512 | 10.27 |
| PA14_10160 | fepD | Ferric enterobactin transport protein FepD | 512 | 9.89 |
| PA14_22270 | Unknown | Possible recombinase | 341.333333 | 9.48 |
| PA14_56920 | inaA | InaA protein | 512 | 9.34 |
| PA14_26940 | Unknown | CHAD domain-containing protein | 512 | 9.32 |
| PA14_40290 | lasA | Protease LasA (EC 3.4.24.-) (Staphylolytic protease) | 512 | 9.26 |
| PA14_14160 | Unknown | Putative acetyltransferase | 512 | 9.03 |
| PA14_48115 | aprD | Alkaline protease secretion protein AprD | 512 | 8.88 |
| PA14_12770 | Unknown | Uncharacterized protein | 512 | 8.86 |
| PA14_68660 | rimK | Probable alpha-L-glutamate ligase (EC 6.3.2.-) | 512 | 8.83 |
| PA14_14040 | rhlB | ATP-dependent RNA helicase RhlB (EC 3.6.4.13) | 10.006317 | 8.59 |
| PA14_31560 | Unknown | Putative transcriptional regulator, LysR family | 172.785066 | 8.58 |
| PA14_32460 | Unknown | Putative transcriptional regulator | 512 | 8.52 |
| PA14_30710 | Unknown | RNA chaperone ProQ | 256 | 8.42 |
| PA14_46020 | Unknown | DTW domain-containing protein | 512 | 8.29 |
| PA14_66150 | Unknown | Uncharacterized protein | 512 | 8.18 |
| PA14_30660 | uvrC | UvrABC system protein C (Protein UvrC) (Excinuclease ABC subunit C) | 512 | 8.16 |
| PA14_66920 | ubiB | Probable protein kinase UbiB (EC 2.7.-.-) (Ubiquinone biosynthesis protein UbiB) | 11.7863626 | 8.11 |
| PA14_64280 | Unknown | Putative branched-chain amino acid ABC transporter, permease protein | 256.5296 | 7.57 |
| PA14_04810 | Unknown | Aldehyde dehydrogenase | 341.333333 | 7.35 |
| PA14_64000 | Unknown | Putative translation initiation factor SUI1 | 512 | 7.31 |
| PA14_06540 | bioC | Malonyl-[acyl-carrier protein] O-methyltransferase (Malonyl-ACP O-methyltransferase) (EC 2.1.1.197) (Biotin synthesis protein BioC) | 512 | 7.28 |
| PA14_39410 | Unknown | Putative acetyltransferase | 512 | 7.19 |
| PA14_31750 | Unknown | Putative acyltransferase | 512 | 6.92 |
| PA14_01670 | Unknown | Putative ATP-binding component of ABC transporter | 512 | 6.89 |
| PA14_49740 | Unknown | Uncharacterized protein | 222.990181 | 6.83 |
| PA14_49480 | Unknown | ABC transporter domain-containing protein | 174.197333 | 6.78 |
| PA14_45520 | Unknown | Putative plasmid partitioning protein | 259.177599 | 6.67 |
| PA14_24590 | Unknown | Uncharacterized protein | 512 | 6.66 |
| PA14_71910 | wbpZ | Glycosyltransferase WbpZ | 512 | 6.57 |
| PA14_56070 | mvaT | Transcriptional regulator MvaT, P16 subunit | 15.482892 | 6.47 |
| PA14_36420 | Unknown | Putative histidine kinase | 512 | 6.45 |
| PA14_35050 | Unknown | Putative protease | 512 | 6.45 |
| PA14_24650 | rmf | Ribosome modulation factor (RMF) | 512 | 6.43 |
| PA14_44280 | rlmM | Ribosomal RNA large subunit methyltransferase M (EC 2.1.1.186) (23S rRNA (cytidine2498-2'-O)-methyltransferase) (23S rRNA 2'-O-ribose methyltransferase RlmM) | 256 | 6.43 |
| PA14_00010 | dnaA | Chromosomal replication initiator protein DnaA | 171.196267 | 6.33 |
| PA14_54770 | Unknown | Uncharacterized protein | 512 | 6.21 |
| PA14_46930 | gltK | Putative permease of ABC transporter | 257.002852 | 6.16 |
| PA14_60650 | Unknown | Uncharacterized protein | 341.333333 | 6.13 |
| PA14_53150 | Unknown | Probable ATP-binding/permease fusion ABC transporter | 512 | 6.05 |
| PA14_24420 | Unknown | DUF58 domain-containing protein | 512 | 6.05 |
| PA14_11730 | Unknown | Possible protein kinase | 256 | 5.79 |
| PA14_18880 | nth | Endonuclease III (EC 4.2.99.18) (DNA-(apurinic or apyrimidinic site) lyase) | 512 | 5.77 |
| PA14_59550 | Unknown | Uncharacterized protein | 512 | 5.74 |
| PA14_35800 | Unknown | Uncharacterized protein | 512 | 5.69 |
| PA14_49930 | Unknown | Uncharacterized protein | 256.5296 | 5.65 |
| PA14_63530 | selB | Selenocysteine-specific elongation factor | 512 | 5.59 |
| PA14_66650 | pilN | Type 4 fimbrial biogenesis protein PilN | 256.2648 | 5.48 |
| PA14_29590 | Unknown | Putative transcriptional regulator | 8.90398237 | 5.47 |
| PA14_25430 | lolC | Putative lipoprotein releasing system, permease protein | 172.078933 | 5.41 |
| PA14_61790 | pth | Peptidyl-tRNA hydrolase (PTH) (EC 3.1.1.29) | 171.593248 | 5.39 |
| PA14_42390 | exsA | Transcriptional regulator ExsA | 512 | 5.23 |
| PA14_30260 | bpt | Aspartate/glutamate leucyltransferase (EC 2.3.2.29) | 512 | 5.22 |
| PA14_14830 | rlmN | Dual-specificity RNA methyltransferase RlmN (EC 2.1.1.192) (23S rRNA (adenine(2503)-C(2))-methyltransferase) (23S rRNA m2A2503 methyltransferase) (Ribosomal RNA large subunit methyltransferase N) (tRNA (adenine(37)-C(2))-methyltransferase) (tRNA m2A37 methyltransferase) | 512 | 5.21 |
| PA14_13680 | Unknown | Putative short-chain dehydrogenase | 512 | 5.18 |
| PA14_40840 | sohB | Putative protease | 14.6310844 | 5.11 |
| PA14_58060 | Unknown | UPF0307 protein PA14_58060 | 512 | 5.09 |
| PA14_11970 | Unknown | Putative 3-methyladenine DNA glycosylase (EC 3.2.2.-) | 512 | 5.07 |
| PA14_08050 | Unknown | Putative tail fiber protein | 512 | 5.01 |
