# Supplementary material for: The Histone H1-Like Protein AlgP Facilitates Even Spacing of Polyphosphate Granules in Pseudomonas aeruginosa
Source: mBio. 2022 Apr 18;13(3):e02463-21. doi: 10.1128/mbio.02463-21 (PMC9239181; doi:10.1128/mbio.02463-21)
Supplement: TABLE S3 [file mbio.02463-21-st003.docx]

**Table S3a: Fluorescence foci summary**

|  | DAPI | | | mApple | | |
| --- | --- | --- | --- | --- | --- | --- |
| Strain | 1-foci | 2-foci | >2-foci | 1-foci | 2-foci | >2-foci |
| WT | 17±2 | 64±2 | 19±4 | - | - | - |
| *algP-mApple* | 14±1 | 55±2 | 32±3 | 5±3 | 53±2 | 41±4 |
| *mApple-algP* | 72±5 | 27±4 | 2±0.5 | 75±4 | 24±4 | 2±0.3 |
| *∆algP* | 77±0.2 | 22±0.2 | 1±0.3 | - | - | - |
| *algP*∆CTD | 71±4 | 28±4 | 2±1 | - | - | - |
| *∆algP P_algP_:algP* | 33±4 | 62±3 | 6±2 | - | - | - |

**Table S3b: Transmission Electron Microscopy Summary Data**

|  | WT | | *∆algP* | | *algP*∆CTD | |
| --- | --- | --- | --- | --- | --- | --- |
|  | 1.5h | 3h | 1.5h | 3h | 1.5h | 3h |
| Granule #/cell | 4.3±2.1 | 2.8±1.1 | 1.3±1.0 | 1.6±0.9 | N/A | 1.6±0.6 |
| Total granular volume/cell  (x10^-3^ µm^3^) | 8.3±3.8 | 14±5.3 | 6.1±5.1 | 17±8.2 | N/A | 16±8.9 |
| Volume of largest granule/cell  (x10^-3^ µm^3^) | 3.9±2.7 | 7.4±2.7 | 5.4±4.7 | 15±7.7 | N/A | 14±7.7 |
| Average granule volume (µm^3^)  (x10^-3^ µm^3^) | 1.9±1.9 | 4.8±3.2 | 4.0±4.4 | 10±9.0 | N/A | 10±8.2 |

**Table S3c: Cell cycle exit**

|  | % >1 origin/cell | | % >0 fork/cell | |
| --- | --- | --- | --- | --- |
|  | 0h | 6h | 0h | 6h |
| WT | 86±2 | 10±6 | 68±18 | 3±2 |
| ∆polyP | 88±5 | 87±4 | 74±5 | 35±3 |
| *∆algP* | 56±4 | 13±5 | 70±8 | 2±2 |
