# Supplementary material for: The Histone H1-Like Protein AlgP Facilitates Even Spacing of Polyphosphate Granules in Pseudomonas aeruginosa
Source: mBio. 2022 Apr 18;13(3):e02463-21. doi: 10.1128/mbio.02463-21 (PMC9239181; doi:10.1128/mbio.02463-21)
Supplement: TEXT S1 [file mbio.02463-21-s0001.docx]

**Supporting Information (SI)**

**The Histone H1-like protein AlgP facilitates even spacing of polyphosphate granules in *Pseudomonas aeruginosa*.**

**Ravi Chawla*, Steven Klupt*, Vadim Patsalo, James R Williamson, Lisa R Racki**

*****Ravi Chawla and Steven Klupt contributed equally to this work. Author order was determined alphabetically

**Affiliations:**

^a^Department of Integrative Structural and Computational Biology, Scripps Research, La Jolla, CA

^b^Department of Immunology and Microbiology, Scripps Research, La Jolla, CA

^†^Correspondence to: [lracki@scripps.edu](mailto:lracki@scripps.edu)

**Supporting Information Appendix**

**SI Text**

**SI Figure and table legends**

**SI Methods**

**SI References**

**Table S1.** Summary of proteomics data. Table is included as a separate .csv file

**Table S2a**. Summary of highly abundant and enriched proteins identified in the pellet.

**Table S2b**. Summary of highly enriched proteins and positively charged proteins identified in granules.

**Table S3a.** Fluorescence foci summary.

**Table S3b.** Transmission Electron Microscopy Summary Data.

**Table S3c.** Cell cycle exit.

**Table S4a.** Strains

**Table S4b.** Plasmids

**Table S4c.** Primers

**SI Text**

**Conservation of AlgP**

AlgP is a widely conserved protein in the *Pseudomonas* genus in proteobacteria. The N-terminal domain of AlgP is present in Pseudomonads like *Pseudomonas putida*, *Pseudomonas fluorescens*, *Pseudomonas syringae*, *Pseudomonas stutzeri* etc., and outside of the *Pseudomonadaceae* family in pathogens like *Acinetobacter baumannii* (*Moraxellaceae* family, *Pseudomonadales* order). An additional database search (see SI methods) revealed that AlgP is present in pathogens like *Klebsiella pneumoniae* (order Enterobacterales). Oddly, we also found an AlgP-like protein outside of proteobacteria in *Streptococcus* (*Streptococcus dysgalactiae* subsp. equisimilis strain NCTC11565 and *Streptococcus pneumoniae*) and *Bacillus* species in the phylum Firmicutes. A constraint based multiple alignment (COBALT) of the representative sequences described above is shown in Figure S4C . While the NTD of the AlgP is highly conserved in these sequences (also See Fig S4D), the histone H1-like CTD exhibits a lot of variability(1). We would like to note, however, that there is a need for more extensive phylogenetic analysis of conservation of the CTD. Low complexity regions are known to commonly give spuriously high BLAST scores that reflect compositional bias rather than significant position by position alignment(2).

**SI FIGURE AND TABLE LEGENDS**

**Figure S1. Extended proteomic panels of proteome from the three biological experiments shown in Figure 1.** (A) Schematic of polyP granule enrichment protocol. (B) Average absolute mass fraction of proteins in the “pellet” and “lysate”, obtained from spectral counting shown in parts per million, as in Fig 1C in the main text, on a linear scale representing the complete and unfiltered proteomic data. (C) Charge of proteins identified in the proteomics analysis plotted against the fold enrichment over the complete charge range (Fig 1E in comparison shows the data for a negative charge cut-off of -75). Figure labels and cut-offs are same as in Figure 1. (D) Absolute mass fraction of proteins in the “pellet” and “lysate”, obtained from spectral counting shown in parts per million. Data are shown for a representative experiment. (E) Enrichment of proteins in the pellet plotted against the abundance of proteins in the pellet for a representative experiment. (F) Charge of proteins identified in a representative proteomics experiment plotted against the calculated fold enrichment (negative charge cut-off for plotting: -75). Figure labels and cut-offs are same as in Figure 1.

**Figure S2. AlgP protein sequence.** (A) The boxed region highlights the 154 residue contiguous repeat domain in the C-terminal domain. Highlighted are 25 perfect KPAA repeats (yellow), interspersed with variants, including 7 KPVA (green), 4 KTAAA (cyan), one KPAV, and two alanine spacers, and two KPAA repeats (yellow) that fall outside of this 154aa contiguous region. (B) Alignment of AlgP and PhaF. Multiple sequence alignment of representative AlgP-like proteins generated using NCBI COBALT tool are shown in C and D. C) Graphical overview of the multiple sequence alignment of complete protein from representative bacterial species. Highly conserved and less conserved amino acid positions based on the relative entropy threshold of the residue are highlighted. Only alignment positions with no gaps are colored. Red indicates highly conserved positions and blue indicates lower conservation. D) Graphical overview of the NTD (amino acids 1-132) from Panel C. Color coding as indicated for C. Details of the strains used for alignment: *Pseudomonas aeruginosa* PA 14, *Pseudomonas aeruginosa* PAO1, *Pseudomonas viridiflava*, *Pseudomonas fluorescens*, *Pseudomonas putida* , *Pseudomonas syringae*, *Pseudomonas stutzeri*, *Acinetobacter baumannii* , *Klebsiella pneumoniae*, *Priestia aryabhattai*, *Streptococcus dysgalactiae* subsp. equisimilis, *Streptococcus pneumoniae* and *Bacillus sp.* TH86.

**Figure S3.** **Effect of AlgP and C-terminus of AlgP on localization of polyP granules during nitrogen starvation and complementation analysis.** (A) Quantification of the fraction of cells in the population with 1, 2, and >2 DAPI and mApple-labeled foci per cell. Each point represents an independent experimental replicate performed on different days, the bar indicates the mean. (B) Representative images of DAPI-stained cells for complementation analysis. (C) Quantification of the fraction of cells in the population with 1, 2, and >2 DAPI-labeled foci per cell for the complementation analysis. One-way ANOVA followed by Tukey HSD multiple comparison test was used to calculate statistical significance, with asterisks showing significant differences as follows: * indicates p< 0.05, ** indicates p< 0.01, *** indicates p< 0.001 **** indicates p < 0.0001. Scale bar: 2µm.

**Figure S4.** **Example of ‘satellite’ granules in cells imaged by TEM.** (A) Two examples of 3h nitrogen-starved WT cells displaying small satellite granules. (B) Three examples of 0h (exponential phase) WT cells. Top and middle cells display nascent granules, but the bottom cell does not. Scale bar: 0.5µm.

**Figure S5.** **Effect of polyP and AlgP on growth.** Optical density (500 nm absorbance) as a function of time for *P. aeruginosa* cultures in MOPS minimal media. Cells were subcultured from overnight cultures where cells were preconditioned in MOPS minimal media.

**Table S1.** **Summary of proteomics data.** The fractional abundance of proteins found in the “pellet” and “lysate” fractions from three independent experiments and their averages are provided in a tabular format.

**Table S2a**. **Summary of highly abundant and enriched proteins identified in the pellet.** The table summarizes the proteins that were both highly abundant in the pellet and highly enriched in the “pellet” fraction. (Abundance cut-off: 5000ppm, Enrichment cut-off:8). These are the proteins found in the upper right quadrant, Figure 1D

**Table S2b**. **Summary of highly enriched proteins and positively charged proteins identified in granules.** The table lists the proteins that were both highly abundant in the pellet and highly positively charged. (The enrichment cut-off was 8 fold, the charge cut-off was +5). This table includes the proteins plotted in the upper right quadrant of Fig 1E that have a charge greater than +5.

**Table S3a. Fluorescence foci summary.** Quantification of percentages of 1, 2, and >2-foci cells in DAPI and mApple channels. Values represent average and standard deviation of between three to six independent experiments performed on different days.

**Table S3b. Transmission Electron Microscopy Summary Data.** Average and standard deviation values calculated from the total population of cells as depicted in Figure 4.

**Table S3c. Cell cycle exit.** Quantification of percentage of cells with >1 origin per cell and >0 DNA replication forks per cell. Values represent average and standard deviation of three independent experiments performed on different days, as shown in Figure 6.

**Table S4a. Strains**

**Table S4b. Plasmids**

**Table S4c. Primers**

### SI METHODS

**Cell growth and harvesting**

*P. aeruginosa* cells were grown as described previously with scale up specific modifications for proteomics noted below(3). WT(PA14) and polyP(LR119) quadruple knockout were streaked from a glycerol stock onto an LB plate and the plate was incubated overnight at 37 °C. A single colony was then inoculated into 25mL complete MOPS minimal media (MMM) and grown to saturation at 37 ºC overnight with shaking at 250 rpm in a 250mL Erlenmeyer flask. Overnight culture (~7mL) was inoculated into 1 L MMM complete media and allowed to grow at 37 ºC to OD500 between 0.4-0.6 in a 2.8L Pyrex Fernbach-style culture flask. The cells were spun down in 1L centrifuge bottles at 5,000 g for 10 min at room temperature (JLA 9.1000 rotor; Avanti J-E centrifuge) and resuspended to an OD_500_ of ~0.4 in 1L low nitrogen MMM media in Pyrex Fernback-style culture flask. At this point, the cultures were incubated back in the 37°C incubator with shaking and allowed to grow for 3h. The cells were then spun in a 1L centrifuge bottle at 5000g for 10min. The supernatant was removed, the pellet resuspended to a volume of 10mL spent medium and aliquoted into Eppendorf tubes (Note:1mL of this cell culture at this step corresponds to 100mL of original cell culture prior to the previous centrifugation step). The Eppendorf tubes were then spun at 5000 g for 5min on a tabletop centrifuge, supernatant discarded and the cell pellets flash frozen in liquid nitrogen. The cell pellets were then stored at -80C till further processing.

**Granule isolation**

Previously generated cell pellets were removed from -80C and incubated in freshly-prepared and chilled lysis buffer. Specifically, the pellet corresponding to each ~200mL cell culture was resuspended in 1mL of lysis buffer and incubated on ice for 15 minutes. The lysis buffer composition was as follows: 200mM NaCl, 35.5mM Na_2_HPO_4_,14.5mM NaH_2_PO_4_, 2mM EDTA, 1X Protease Inhibitor Mix, 0.2mg/mL Lysozyme, 1% Triton X-100. The resuspended solution became extremely viscous upon incubation and was sonicated with a microtip sonicator (Qsonica Q700; 70% power, 3 minutes total [10 seconds on, 10 seconds off]) on a CoolRack or ice slurry. After sonication, about ~30µL of this lysate was flash frozen in liquid N_2_ and stored at -80°C. This fraction was labeled the “lysate” fraction used for proteomic analysis. To the remaining lysate, 1µL of 1M MgCl_2_, 1µL DNAse I (2000UµL) and 1uL Benzonase (250U/µL) was added per mL of the lysate and the solution was incubated for 30 minutes at room temperature. Next, about ~500uL of the lysate from the previous step was loaded onto 7mL of pre-chilled Percoll gradient solution in ultracentrifuge tubes (Beckman Coulter #355630). Percoll gradient composition was follows: 90% Percoll (Cytiva #17089101), 200 mM NaCl, 35.5mM Na_2_HPO_4_, 14.5mM NaH_2_PO_4_ and 3mM MgCl_2_. Ultracentrifugation was performed on a pre-chilled rotor (50TI) at 21,300 rpm for 15 minutes in an ultracentrifuge (Beckman Coulter Optima L-80 XF) maintained at 4°C. Upon ultracentrifugation a Percoll-encased polyP pellet was clearly visible in the bottom of the tube for wildtype cells and absent in the case of quadruple polyP mutant. The supernatant was removed from the ultracentrifuge tube using a serological pipette leaving behind the Percoll-encased pellet. The Percoll-encased pellet was resuspended in 1mL of dilution buffer (200 mM NaCl, 35.5mM Na_2_HPO_4_, 14.5mM NaH_2_PO_4_, 3mM MgCl_2_). The resuspended sample was then centrifuged in a table-top microcentrifuge at 10,000 g for 2min and the supernatant discarded. The pellet was then resuspended in 200uL of dilution buffer and washing was repeated once more to remove the Percoll. The samples at this step were labeled as “pellet” samples, flash frozen in liquid N_2_ and stored at -80°C.

**Proteomics Sample Preparation**

Percoll pellet biomass was resuspended in 100 µL HPLC-grade water and protein was precipitated by methanol–chloroform extraction(4). Insoluble material was pelleted by centrifugation at 17,000 *g* and re-solubilized in 40 µL of freshly-made 8M urea buffered by 100 mM Tris-HCl, pH 7.5. Disulfides were reduced with 10 mM DTT (30 min at 37°C), alkylated with 40 mM iodoacetamide (30 min at 37C). Reaction was quenched with 20 mM DTT (30 min at 37°C).

For compatibility with tryptic digestion, reaction volume was diluted with 50 mM Tris-HCl, 10 mM CaCl_2_ to 200 µL (final concentration of 1.6M Urea). Cellular protein was digested for 16 hours at 37C with 2 µg porcine pancreatic trypsin (Thermo), and followed by an booster dose of 1 ug trypsin for 4 hours. Peptides were de-salted using PepClean columns (Thermo), dried using a SpeedVac, and re-suspended in 20 µL of LC-MS solvent A (Honeywell, 0.1% formic acid in water) prior to analysis.

**Proteomics Data Acquisition**

Tryptic peptides (2-3 µL, approximately 1-2 µg based on absorbance) were injected onto an Eksigent EKSPERT NanoLC 425 chromatography system operating in trap-elute mode. Peptides were eluted from a SCIEX ChromXP analytical C18 reverse-phase nanoflow column (3 μm, 120Å, 150 x 0.3 mm) over the course of a 120-minute linear ramp gradient (5-35% of 0.1% formic acid in acetonitrile) at 300 nL/min. A SCIEX TripleTOF 5600 operating in DDA mode was used for data acquisition in positive polarity mode. An MS1 survey scan (250 ms, 400-1250 Th, high-resolution) was followed by 20 product ion scans (150 ms, 100-1500 Th, high-sensitivity). Ions with a charge of +2 to +4 exceeding 100 counts-per-second were subjected to fragmentation. Collision energy was set to ‘rolling’. Former targets ions were excluded for 15 sec after one occurrence.

**Proteomics Data Analysis**

Vendor (.WIFF format) data files were converted with the SCIEX MS Data Converter (Beta 1.3) to mzXML format in centroid mode. The trans-Proteomic Pipeline software suite was used to search the data with X!TANDEM against a UniProt *Pseudomonas aeruginosa (strain UCBPP-PA14)*  database (proteome UP000000653, retrieved October 2019) supplemented with common contaminants, enzymes and reversed peptide decoy sequences(5, 6). The peptide-spectrum match tolerances were: 50 ppm and 100 ppm for the precursor and product ions. PeptideProphet and iProphet were used to combine the peptide–spectrum matches across multiple samples and SpectraST was used to generate spectral libraries and collate search results(7).

*Absolute quantification.* To estimate the (g/g) absolute mass fraction of a protein in a given sample, we used the spectral counting technique(8). For a protein, its mass fraction abundance was tabulated by dividing the total number its peptide-spectrum matches (PSM) by the total of all 14N PSMs in the sample. The abundances of proteins from three independent experiments (and the averages, refer to the section below) are reported in Table S1.

**Software used for proteomic analyses.** Further proteomic data processing and analysis was performed in Python (CPython 3.7.7, IPython 7.21.0) with NumPy version 1.19.2 and Pandas version 1.2.3 using Jupyter notebook (Jupyerlab version 3.0.11). Data was plotted with Bokeh version 2.3.0 and the figures were assembled in Adobe Illustrator. Enrichment of a protein in our proteomics screen was defined as the ratio of the abundance of protein in the pellet to the abundance in the lysate. This definition leads to an “infinite” enrichment when abundance of a protein in the lysate is zero and to computationally handle these infinite values in Pandas DataFrame a value of 512 (2^9^ i.e., log_2_ fold change=9) was assigned. The averaging of proteins from three separate experiments was performed using the built-in mean function of the Pandas Dataframe with the ‘skipna’ parameter set to ‘True’ to exclude the NA/null values when computing the result. An outcome of this selection is that the proteins with infinite enrichment in one or more experiments show up as a band or clusters, an artifact we termed “banding.” In our current analysis we have not pursued the banding pattern, but in future it would be curious to probe if some of the proteins exhibiting the banding pattern could be part of the interactome of the polyphosphate granule that transiently and/or weakly associated. The charges of the protein (at pH 7) on PA14 proteome were obtained from UniPort (UP000014183; date accessed February 18^th^, 2020) and integrated it in Jupyter notebook analysis pipeline by a Panda DataFrame merge operation on the protein locus ID. An abundance value of >5000 ppm and enrichment value of 8 (2^3^ i.e., log_2_ fold change = 3) were, respectively, used as cut-offs for “high” abundance and enrichment in our analysis discussed in Figure 1 (also see Table S2A,B).

**Conservation Determination**

The conservation was determined using search of curated databases on National Center for Biotechnology Information (NCBI). The NCBI curated protein family model based on the Hidden Markov Model (HMM) of the N-terminal domain of AlgP protein (NCBI HMM accession NF038178.1) identifies AlgP to be present in Pseudomonads like *Pseudomonas putida*, *Pseudomonas fluorescens*, *Pseudomonas syringae*, *Pseudomonas stutzeri* etc. An additional database search, using the NCBI BLAST-P tool, reveals that AlgP to be present outside of the *Pseudomonadaceae* family in pathogens like *Acinetobacter baumannii* (*Moraxellaceae* family, *Pseudomonadales* order). Our search also revealed that AlgP is present in pathogens like *Klebsiella pneumoniae* (order Enterobacterales). Oddly, we also found an AlgP-like protein outside of proteobacteria in *Streptococcus* (*Streptococcus dysgalactiae* subsp. equisimilis strain NCTC11565) and *Streptococcus pneumoniae*) and *Bacillus* species in the phylum Firmicutes. A constraint based multiple alignment (COBALT) of the representative sequences described above is shown in Figure S2C . While the NTD of the AlgP is highly conserved in these sequences (also See Fig S2D), the histone H1-like CTD exhibits a lot of variability.

Extensive lysine-rich repeat-containing proteins with histone-like sequence characteristics have previously been reported for *Chlamydia trachomatis*, *Coxiella burnetii*, *Bordetella pertussis*, *Streptomyces coelicolor*, *Salmonella typhimurium* and *E. coli(1)*. We would like to note, however, that there is a need for a more comprehensive sequence analysis of bacterial proteomes to look for proteins with domains similar to the C-terminal domain of AlgP. Low complexity regions are known to commonly give spuriously high BLAST scores that reflect compositional bias rather than significant position by position alignment(2).

**Sequence Alignment**

Multiple sequence alignment of the representative proteins was generated using NCBI’s constraint based multiple alignment (COBALT) tool(9). The graphical overview of the multiple sequence alignment of the proteins in Fig S4C depict highly conserved and less conserved amino acid positions based on the relative entropy threshold of the residue are highlighted. Only alignment positions with no gaps are colored. Red indicates highly conserved positions and blue indicates lower conservation. Following parameter set were used to for COBALT: Alignment Parameters: Gap penalties -11,-1; End-Gap penalties -5,-1. CDD Parameters: Use RPS BLAST on; Blast E-value: 0.003; Find Conserved columns and Recompute: on. Query Clustering Parameters: Use query clusters: on; Word Size: 4; Max cluster distance: 0.8; Alphabet: Regular. The details of the strain and sequences used for alignment are as follows: *Pseudomonas aeruginosa* PA14 (PA14_69370), *Pseudomonas aeruginosa* PAO1 ( PA5253) *Pseudomonas viridiflava* (Accession: WP_162873968.1), *Pseudomonas fluorescens* (Accession: WP_150640702.1),  *Pseudomonas putida* (Accession: WP_225136748.1), *Pseudomonas syringae* (Accession: WP_047572816.1), *Pseudomonas stutzeri* (Accession: WP_011911763.1), *Acinetobacter baumannii* (Accession: SVK36065.1), *Klebsiella pneumoniae* (Accession: SVJ50573.1 ), *Priestia aryabhattai* (Accession: QPN45735.1), *Streptococcus dysgalactiae* subsp. Equisimilis (Accession: VTS46242.1), *Streptococcus pneumoniae* (Accession: CJK71812.1) and *Bacillus sp.* TH86 (Accession: MBK5302748.1).

**Fluorescence microscopy**

All live cell imaging was acquired with a Nikon Ti2-E inverted microscope with perfect focus and the following other hardware: Objective: Plan apochromat phase contrast 100X oil immersion objective, N.A. 1.45, Illumination Source: For brightfield, a white LED, for fluorescence, the Spectra X Light Engine with a 470nm LED (Lumencor). Camera: Prime 95B sCMOS with 11 µm x 11 µm pixel area (Photometrics). Image acquisition was controlled using Nikon Elements. The following parameters were used: For phase contrast: 75% light intensity, 100ms exposure time, gain = 1.0. For mNeon, GFP, and SybrGreen imaging: 100% light intensity from the 470nm LED, 100ms exposure time and a GFP filter cube (466/40nm excitation filter, 525/50nm emission filter, 495nm dichroic mirror, Semrock), gain = 1.0. For mCherry and mApple: 100% light intensity from the 555nm LED, 100ms exposure time and Texas Red filter cube (562/40nm excitation filter, 641/75nm emission filter, 593nm dichroic mirror, Semrock), gain = 1.0. For DAPI imaging of polyP: 100% light intensity from the 395nm LED, 100ms exposure time and a custom filter cube (415/20nm excitation filter, 555/10nm emission filter, 425nm long pass dichroic mirror, Semrock), gain = 1.0.

**Image analysis**

For fluorescent foci detection using MicrobeTracker’s spotFinderZ, the following parameters were used: Expand cell, px = 1, low cutoff, px = 1, high cutoff, px = 3, min filtered height, i.u =0, shift limit = 0.01, Fit area size, px = 6, resize, times = 1, remove ridges (checked), scale factor = 1, max width squared px^2 = 9.4242, min width squared, px^2 = 3.4021, min height, i.u. = 0.0021186, max rel. sq. error = 45.9292, Max var/sq. height ratio = 1.2216, min filtered/fitted ratio = 0.027113. Spot detection was manually corrected with spotFinderM.

For the scatter plots in Figure 2E and 2F to show the correlation of spot positions in the DAPI and mCherry channels, spots in each channel were first ordered by their position on the long axis of the cell and their normalized relative position was determined using the Matlab script SpotOrdering.m on the cellList.mat files generated by MicrobeTracker. For Figure 2E, the Matlab script spotlister2.m was then used to generate an inclusive list of all cells that had 2 foci in at least one of the channels, and to calculate their relative positions. Briefly, spotlister2.m does the following: For cells which had 2 foci in both channels, the distance between the first focus in the DAPI channel with both foci in the mApple channel was calculated, and whichever focus in the mCherry channel was closer was paired with it in the list. The other two spots are then paired. For cells that had 2 foci in the DAPI channel, but 1 focus in the mApple channel, the mApple focus was paired with its closest DAPI focus. The unpaired DAPI focus was saved and plotted as having a position of zero in the mApple channel. For cells that had 2 foci in the DAPI channel and no foci in the mApple channel, both foci were plotted as having a position of zero in the mApple channel. For cells that had 2 foci in the DAPI channel, but more than 2 in the mApple channel, the distance between each mApple focus and each of the 2 DAPI foci was calculated, and the closest mApple focus to each of the DAPI foci were paired. The extra mApple foci that were not paired are saved in the list and plotted as having a position of zero in the DAPI channel. For cells that had 2 foci in the mApple channel, but 1 focus in the DAPI channel, the DAPI focus was paired with its closest mApple focus, and the unpaired mApple focus was saved and plotted as having a position of zero in the DAPI channel. For cells that had 2 foci in the mApple channel and no foci in the DAPI channel, both foci were plotted as having a position of zero in the DAPI channel. For cells that had 2 foci in the mApple chanel, but more than 2 in the DAPI channel, the distance between each DAPI focus and each of the 2 mApple foci was calculated, and the closest DAPI focus to each of the mApple foci were paired. The extra DAPI foci that were not paired are saved in the list and plotted as having a position of zero in the mApple channel. A similar procedure was performed for Figure 2F using the Matlab script spotlister3.m for 3-foci cells. These MATLAB scripts are provided at Zenodo.org: 10.5281/zenodo.6172994

**Data Availability.**

The mass spectrometry proteomics data have been deposited and available to ProteomeXchange Consortium via the UCSD's MassIVE repository with the accession codes: MassIVE: MSV000087218 and ProteomeXchange: PXD025444. Plasmid maps, raw light and electron microscopy data, and MATLAB code used in image analysis are available at zenodo.org: 10.5281/zenodo.6172994 and 10.5281/zenodo.6172996

**Strain construction**

Strains, plasmids, and primers used in this study are listed in Tables S4a-c respectively.

### Strains

All unmarked deletion strains, and strains in which endogenous proteins are replaced by fluorescent chimeras, were generated by triparental conjugation with *P. aeruginosa* UCBPP-PA14, and then merodiploids were selected as described previously on VBMM medium (3 g/L trisodium citrate, 2 g/L citric acid, 10g/L K2HO4, 3.5 g/L NaNH4PO4, 1mM MgSO4, 100uM CaCl2, pH 7) containing 100 ug/mL gentamicin(10). Counterselection for homologous recombination events removing the endogenous copy of the gene in question was then performed on LB plates without NaCl and containing 300mM sucrose, followed by PCR verification. All strains with insertions at the *att*Tn7 site were generated by tetraparental conjugation with *P. aeruginosa* UCBPP-PA14, and then exconjugants were selected on VBMM medium, and verified by PCR(10).

### Plasmids

All plasmids were generated using either Gibson cloning or the yeast gap repair method of

homologous recombination by *Saccharomyces cerevisiae*(11–13). Inserts were generated by PCR, or from synthetic gBlock gene fragments (Integrated DNA Technologies). Plasmids pLREX79, pLREX120, pLREX120, pLREx121, pLREX124, and pLREX125 are derivatives of suicide vector pMQ30 (2), generated by amplifying ~1 kb of sequence upstream and downstream of the target gene from *P. aeruginosa* genomic DNA. Plasmid pLREX132 is a derivative of the pUC18T-mini-Tn7T-Gm suicide vector.

**pLREX79** *[ppk2A::ppk2A-20aa-mNeonGreen]* was created by yeast homologous recombination between digested plasmid pLREX9 and gBlock6. Plasmid pLREX9 [*ppk2A::ppk2A-mCherry*] was cut with NotI and XmaI to remove mCherry. Plasmid confirmed by Sanger sequencing.

**pLREX120** *[∆algP*] was created by a Gibson assembly consisting of pMQ30 cut with HindIII and KpnI and 2 fragments: (1) 815bp PCR product of template *P. aeruginosa* PA14 genomic DNA, primers LRPR894F and LRPR912R, (2) 540bp PCR product of template *P. aeruginosa* PA14 genomic DNA, primers LRPR909F and LRPR899R. Plasmid confirmed by Sanger sequencing.

**pLREX121** [*algP∆CTD*] was created by Gibson assembly consisting of pMQ30 cut with HindIII and KpnI and 2 fragments: (1) 1286bp PCR product of template *P. aeruginosa* PA14 genomic DNA, primers LRPR894F and LRPR933R, (2) 543bp PCR product of template *P. aeruginosa* PA14 genomic DNA, primers LRPR932F and LRPR899R. Plasmid confirmed by Sanger sequencing.

**pLREX124** [*algP::mApple-algP*] was created by Gibson assembly consisting of pMQ30 cut with HindIII and KpnI and 3 fragments: (1) 818bp PCR product of template *P. aeruginosa* PA14 genomic DNA, primers LRPR894F and LRPR904R, (2) 772bp PCR product of template gBlock4, primers LRPR905F and LRPR906R, and (3) 1594bp PCR product of template *P. aeruginosa* PA14 genomic DNA, primers LRPR907F and LRPR899R. Plasmid confirmed by Sanger sequencing.

**pLREX125** [*algP::algP-mApple*] was created by Gibson assembly consisting of pMQ30 cut with HindIII and KpnI and 3 fragments: (1) 1886bp PCR product of template *P. aeruginosa* PA14 genomic DNA, primers LRPR894F and LRPR901R, (2) 771bp PCR product of template gBlock4, primers LRPR900F and LRPR903R, and (3) 535bp PCR product of template *P. aeruginosa* PA14 genomic DNA, primers LRPR898F and LRPR899R. Plasmid confirmed by Sanger sequencing.

**pLREX132** [*P_algP_:algP*]was created by Gibson assembly consisting of pUC18T-mini-Tn7T-Gm cut with HindIII and KpnI and 1 fragment: a 1181bp PCR product of template *P. aeruginosa* PA14 genomic DNA and primers LRPR956F and LRPR955R. This fragment contains the *algP* coding sequence and the 125 bp intergenic region upstream of algP containing the putative previously identified promoter sequence ’CGAACCCGTTGGCGAGAGGGGGTTTGCGGGTCTAGTATGGGCGCAACCAC’ from *P. aeruginosa* PA14 genomic DNA(14, 15).

**Sequences of gBlocks, fluorescent proteins, and linkers**

**gBlock4:**

(***bold/italicized sequence is mApple,*** GenBank: DQ336160.2, codon optimized for *Pseudomonas aeruginosa****)***

CGAGGAGGACGAGAAGGTCTACGCCGAGGCGGCCGCCGCGCCGGGCCACGCGAACCTGGATATCCCGGCCCTCGAGGGGTCCGGTCAGGGACCGGGATCCGGCCAAGGGTCCGGC***ATGGTGTCGAAGGGCGAGGAAAACAATATGGCCATCATCAAAGAGTTCATGCGGTTCAAGGTCCATATGGAGGGGTCGGTCAATGGGCACGAGTTCGAGATCGAAGGCGAAGGCGAGGGGCGGCCGTATGAGGCGTTCCAGACCGCGAAGCTGAAGGTCACGAAGGGGGGGCCGCTCCCCTTCGCGTGGGACATCCTCTCCCCCCAATTCATGTATGGCTCCAAAGTCTACATCAAGCATCCGGCCGATATCCCCGATTATTTCAAGCTGAGCTTCCCCGAGGGCTTCCGCTGGGAACGGGTCATGAATTTCGAAGATGGCGGGATCATCCACGTGAACCAAGATAGCAGCCTCCAAGATGGCGTGTTCATCTATAAGGTCAAACTGCGCGGGACGAATTTCCCCTCCGATGGGCCCGTGATGCAGAAAAAAACGATGGGCTGGGAAGCCAGCGAGGAACGCATGTATCCCGAAGACGGCGCCCTGAAGTCGGAGATCAAAAAACGGCTCAAGCTGAAAGATGGGGGCCACTATGCGGCGGAGGTGAAAACCACGTACAAGGCGAAAAAGCCCGTGCAACTCCCCGGCGCGTACATCGTGGACATCAAACTCGATATCGTGAGCCACAATGAGGACTATACCATCGTCGAGCAGTATGAACGCGCCGAAGGCCGGCACTCGACCGGGGGGATGGACGAACTCTATAAATAA***GGCGGGCGGTCGCGCCAACGAAAACGCCCGGGGCGCTTTCGCGCTCCGGGCGTCCC

**gBlock6:**

(underlined sequence is 20aa Linker 1, ***bold/italicized sequence is mNeonGreen)***

CGAGGAGGACGAGAAGGTCTACGCCGAGGCGGCCGCCGCGCCGGGCCACGCGAACCTGGATATCCCGGCCGGATCCGGGCAGGGACCGTCTGGCCAGGGATCGGGGCCAGGATCAGGTCAAGGCTCCGGT***ATGGTTTCGAAAGGAGAGGAGGATAATATGGCTAGCCTCCCAGCGACCCACGAACTGCATATTTTTGGCAGCATTAATGGCGTTGACTTTGATATGGTGGGGCAGGGAACAGGGAACCCTAACGATGGCTATGAGGAGCTCAATCTCAAGAGTACAAAAGGAGATTTGCAATTTTCACCTTGGATCCTGGTTCCGCATATTGGCTACGGCTTTCATCAATACTTGCCTTATCCGGACGGCATGTCCCCGTTCCAAGCTGCGATGGTGGATGGTTCTGGGTACCAGGTGCACCGTACTATGCAGTTTGAGGACGGTGCCTCACTGACGGTCAACTATAGATATACTTATGAAGGCTCACACATTAAGGGTGAGGCCCAAGTTAAAGGAACAGGGTTTCCTGCGGATGGACCGGTAATGACAAACAGTTTAACCGCTGCGGACTGGTGTCGCTCGAAAAAAACATACCCAAACGATAAAACGATCATCTCGACCTTCAAATGGAGCTATACTACGGGCAACGGCAAACGCTATCGTTCCACAGCACGCACGACTTATACGTTTGCTAAACCGATGGCCGCAAACTACCTCAAAAATCAACCTATGTACGTGTTCAGAAAAACCGAGTTAAAACATTCAAAAACGGAACTTAATTTTAAAGAGTGGCAAAAGGCGTTTACAGACGTGATGGGTATGGATGAACTCTATAAGTGA***GGCGGGCGGTCGCGCCAACGAAAACGCCCGGGGCGCTTTCGCGCTCCGGGCGTCCC

**mNeonGreen:**

ATGGTTTCGAAAGGAGAGGAGGATAATATGGCTAGCCTCCCAGCGACCCACGAACTGCATATTTTTGGCAGCATTAATGGCGTTGACTTTGATATGGTGGGGCAGGGAACAGGGAACCCTAACGATGGCTATGAGGAGCTCAATCTCAAGAGTACAAAAGGAGATTTGCAATTTTCACCTTGGATCCTGGTTCCGCATATTGGCTACGGCTTTCATCAATACTTGCCTTATCCGGACGGCATGTCCCCGTTCCAAGCTGCGATGGTGGATGGTTCTGGGTACCAGGTGCACCGTACTATGCAGTTTGAGGACGGTGCCTCACTGACGGTCAACTATAGATATACTTATGAAGGCTCACACATTAAGGGTGAGGCCCAAGTTAAAGGAACAGGGTTTCCTGCGGATGGACCGGTAATGACAAACAGTTTAACCGCTGCGGACTGGTGTCGCTCGAAAAAAACATACCCAAACGATAAAACGATCATCTCGACCTTCAAATGGAGCTATACTACGGGCAACGGCAAACGCTATCGTTCCACAGCACGCACGACTTATACGTTTGCTAAACCGATGGCCGCAAACTACCTCAAAAATCAACCTATGTACGTGTTCAGAAAAACCGAGTTAAAACATTCAAAAACGGAACTTAATTTTAAAGAGTGGCAAAAGGCGTTTACAGACGTGATGGGTATGGATGAACTCTATAAGTGA

**mApple:**

ATGGTGTCGAAGGGCGAGGAAAACAATATGGCCATCATCAAAGAGTTCATGCGGTTCAAGGTCCATATGGAGGGGTCGGTCAATGGGCACGAGTTCGAGATCGAAGGCGAAGGCGAGGGGCGGCCGTATGAGGCGTTCCAGACCGCGAAGCTGAAGGTCACGAAGGGGGGGCCGCTCCCCTTCGCGTGGGACATCCTCTCCCCCCAATTCATGTATGGCTCCAAAGTCTACATCAAGCATCCGGCCGATATCCCCGATTATTTCAAGCTGAGCTTCCCCGAGGGCTTCCGCTGGGAACGGGTCATGAATTTCGAAGATGGCGGGATCATCCACGTGAACCAAGATAGCAGCCTCCAAGATGGCGTGTTCATCTATAAGGTCAAACTGCGCGGGACGAATTTCCCCTCCGATGGGCCCGTGATGCAGAAAAAAACGATGGGCTGGGAAGCCAGCGAGGAACGCATGTATCCCGAAGACGGCGCCCTGAAGTCGGAGATCAAAAAACGGCTCAAGCTGAAAGATGGGGGCCACTATGCGGCGGAGGTGAAAACCACGTACAAGGCGAAAAAGCCCGTGCAACTCCCCGGCGCGTACATCGTGGACATCAAACTCGATATCGTGAGCCACAATGAGGACTATACCATCGTCGAGCAGTATGAACGCGCCGAAGGCCGGCACTCGACCGGGGGGATGGACGAACTCTATAAATAA

**20aa linker 1**

ggatccgggcagggaccgtctggccagggatcggggccaggatcaggtcaaggctccggt

**SI REFERENCES**

1. Kasinsky HE, Lewis JD, Dacks JB, Ausló J. 2001. Origin of H1 linker histones. The FASEB Journal 15:34–42.

2. Tatusova TA, Madden TL. 1999. BLAST 2 Sequences, a new tool for comparing protein and nucleotide sequences. FEMS Microbiology Letters 174:247–250.

3. Racki LR, Tocheva EI, Dieterle MG, Sullivan MC, Jensen GJ, Newman DK. 2017. Polyphosphate granule biogenesis is temporally and functionally tied to cell cycle exit during starvation in *Pseudomonas aeruginosa*. Proc Natl Acad Sci USA 114:E2440–E2449.

4. Wessel D, Flügge UI. 1984. A method for the quantitative recovery of protein in dilute solution in the presence of detergents and lipids. Anal Biochem 138:141–143.

5. Deutsch EW, Mendoza L, Shteynberg D, Farrah T, Lam H, Tasman N, Sun Z, Nilsson E, Pratt B, Prazen B, Eng JK, Martin DB, Nesvizhskii AI, Aebersold R. 2010. A guided tour of the Trans-Proteomic Pipeline. Proteomics 10:1150–1159.

6. Craig R, Beavis RC. 2004. TANDEM: matching proteins with tandem mass spectra. Bioinformatics 20:1466–1467.

7. Lam H, Deutsch EW, Eddes JS, Eng JK, Stein SE, Aebersold R. 2008. Building Consensus Spectral Libraries for Peptide Identification in Proteomics. Nat Methods 5:873–875.

8. Gao J, Friedrichs MS, Dongre AR, Opiteck GJ. 2005. Guidelines for the Routine Application of the Peptide Hits Technique. J Am Soc Mass Spectrom 16:1231–1238.

9. Papadopoulos JS, Agarwala R. 2007. COBALT: constraint-based alignment tool for multiple protein sequences. Bioinformatics 23:1073–1079.

10. Choi K-H, Schweizer HP. 2006. mini-Tn7 insertion in bacteria with single attTn7 sites: example Pseudomonas aeruginosa. Nat Protoc 1:153–161.

11. Oldenburg KR, Vo KT, Michaelis S, Paddon C. 1997. Recombination-mediated PCR-directed plasmid construction in vivo in yeast. Nucleic Acids Res 25:451–452.

12. Shanks RMQ, Caiazza NC, Hinsa SM, Toutain CM, O’Toole GA. 2006. Saccharomyces cerevisiae-Based Molecular Tool Kit for Manipulation of Genes from Gram-Negative Bacteria. Appl Environ Microbiol 72:5027–5036.

13. Gibson DG, Young L, Chuang R-Y, Venter JC, Hutchison CA, Smith HO. 2009. Enzymatic assembly of DNA molecules up to several hundred kilobases. Nat Methods 6:343–345.

14. Kato J, Misra TK, Chakrabarty AM. 1990. AlgR3, a protein resembling eukaryotic histone H1, regulates alginate synthesis in Pseudomonas aeruginosa. Proc Natl Acad Sci U S A 87:2887–2891.

15. Konyecsni WM, Deretic V. 1990. DNA sequence and expression analysis of algP and algQ, components of the multigene system transcriptionally regulating mucoidy in Pseudomonas aeruginosa: algP contains multiple direct repeats. J Bacteriol 172:2511–2520.
