# Supplementary material for: The Histone H1-Like Protein AlgP Facilitates Even Spacing of Polyphosphate Granules in Pseudomonas aeruginosa
Source: mBio. 2022 Apr 18;13(3):e02463-21. doi: 10.1128/mbio.02463-21 (PMC9239181; doi:10.1128/mbio.02463-21)
Supplement: TABLE S4 [file mbio.02463-21-st004.docx]

**Table S4a: Strains**

| Name | Genotype | Source |
| --- | --- | --- |
| LR31; DKN263 | *P. aeruginosa* UCBPP-PA14 |  |
| DKN303 | *E. coli* DH5α, pMQ30 | (12) |
| DKN1297 | *E. coli* DH5α (F− **Δ**(argF-lac)169 **Φ**80dlacZ58(**Δ**M15) glnV44(AS) **λ**− rfbC1 gyrA96(NalR) recA1 endA1  spoT1 thi-1 hsdR17 deoR), pUC18R6K-mini-Tn7T-Gm | (10) |
| DKN1298 | SM10, pTNS1 | (10) |
| DKN1299 | HB101  (F− **λ**− **Δ**(gpt-proA)62 leuB6 glnV44(AS) araC14 galK2(Oc) lacY1 **Δ**(mcrC-mrr) rpsL20(StrR)  xylA5 mtl-1 recA13 hsdS20), pRK2013  pRK2013 has a ColE1 replicon and carries the RK2 tra genes and Tn903 (which is KanR) | (10) |
| LR135; DKN1729 | *P. aeruginosa* UCBPP-PA14 *∆ppk1 ∆ppk2B* *∆ppk2C*; deletion of PA14_69230, PA14_33240, and PA14_19410 in DKN263 | (3) |
| LR79; DKN1730 | *P. aeruginosa* UCBPP-PA14 *∆ppk2A ∆ppk2B* *∆ppk2C*; deletion of PA14_01730, PA14_33240, and PA14_19410 in DKN263 | (3) |
| LR119; DKN1731 | *P. aeruginosa* UCBPP-PA14 *∆ppk1 ∆ppk2A ∆ppk2B* *∆ppk2C*; deletion of PA14_69230, PA14_01730, PA14_33240, and PA14_19410 in DKN263 | (3) |
| LR177; DKN1736 | *P. aeruginosa* UCBPP-PA14 *∆ppk1 ∆ppk2A ∆ppk2B ∆ppk2C* attTn7:: mini-Tn7T-Gm^R^ *P_ssb_:ssb-mCherry* | (3) |
| LR229; DKN1762 | *E. coli* TOP10(F– mcrA Δ(mrr-hsdRMS-mcrBC) Φ80lacZΔM15 ΔlacX74 recA1 araD139 Δ(ara leu) 7697 galU galK rpsL (StrR) endA1 nupG), pLREX62 | (3) |
| LR231 | *P. aeruginosa* UCBPP-PA14  *∆ppk1 ∆ppk2a ∆ppk2b ∆ppk2c*  *att*Tn7::mini-Tn7T-Gm^R^ ParS^pMT1^  *P_ssb_:ssb-mCherry gfp-parB^pMT1^* | This study |
| LR238 | *P. aeruginosa* UCBPP-PA14  *att*Tn7::mini-Tn7T-Gm^R^ ParS^pMT1^  *P_ssb_:ssb-mCherry gfp-parB^pMT1^* | This study |
| LR322 | *E. coli* TOP10(F– mcrA Δ(mrr-hsdRMS-mcrBC) Φ80lacZΔM15 ΔlacX74 recA1 araD139 Δ(ara leu) 7697 galU galK rpsL (StrR) endA1 nupG), pLREX79 | This study |
| LR457 | *E. coli* TurboCells^TM (^F- recA1 endA1 hsdR17 supE44 thi-1 gyrA96 relA1 φ80lacZΔM15 Δ(lacZYAargF)  U169), pLREX120 | This study |
| LR458 | *E. coli* TurboCells^TM (^F- recA1 endA1 hsdR17 supE44 thi-1 gyrA96 relA1 φ80lacZΔM15 Δ(lacZYAargF)  U169), pLREX121 | This study |
| LR461 | *E. coli* TurboCells^TM (^F- recA1 endA1 hsdR17 supE44 thi-1 gyrA96 relA1 φ80lacZΔM15 Δ(lacZYAargF)  U169), pLREX124 | This study |
| LR462 | *E. coli* TurboCells^TM (^F- recA1 endA1 hsdR17 supE44 thi-1 gyrA96 relA1 φ80lacZΔM15 Δ(lacZYAargF)  U169), pLREX125 | This study |
| LR467 | *P. aeruginosa* UCBPP-PA14 *∆algP;* deletion of PA14_69370 | This study |
| LR469 | *P. aeruginosa* UCBPP-PA14 *algP*∆CTD*;* truncation of 195 c-terminal amino acids (157-352) of AlgP (PA14_69370) | This study |
| LR471 | *P. aeruginosa* UCBPP-PA14 *algP::mApple-algP*; replacement of AlgP with chimeric fusion mApple-AlgP | This study |
| LR477 | *P. aeruginosa* UCBPP-PA14 *∆ppk1 ∆ppk2A ∆ppk2B ∆ppk2C* algP::algP-mApple; deletion of PA14_69230, PA14_01730, PA14_33240, and PA14_19410, replacement of algP (PA14_69370) with chimeric fusion algP-mApple | This study |
| LR491 | *P. aeruginosa* UCBPP-PA14 *algP::algP-mApple* ppk2A::ppk2A-mNeonGreen; replacement of AlgP (PA14_69370) with chimeric fusion AlgP-mApple, replacement of Ppk2A (PA14_01730) with chimeric fusion Ppk2A-mNeonGreen | This study |
| LR498 | *P. aeruginosa* UCBPP-PA14 *∆ppk2a ∆ppk2b ∆ppk2c* *algP::algP-mApple*; deletion of PA14_01730, PA14_33240, and PA14_19410, replacement of AlgP (PA14_69370) with chimeric fusion AlgP-mApple | This study |
| LR500 | *P. aeruginosa* UCBPP-PA14 *∆algP* (PA14_69370) attTn7:: mini-Tn7T-Gm^R^ ParS *^pMT1^* *P_ssb_:ssb-mCherry gfp-parB^pMT^* | This study |
| LR501 | *P. aeruginosa* UCBPP-PA14 *∆algP* (PA14_69370) attTn7:: mini-Tn7T-Gm^R^ *P_algP_:algP;*algP complementation | This study |
| LR502 | *P. aeruginosa* UCBPP-PA14 *algP::algP-mApple*; replacement of *algP* with chimeric fusion *algP-mApple* | This study |
| LR503 | *E. coli* TurboCells^TM (^F- recA1 endA1 hsdR17 supE44 thi-1 gyrA96 relA1 φ80lacZΔM15 Δ(lacZYAargF)  U169), pLREX132 | This study |
| LR504 | *P. aeruginosa* UCBPP-PA14 *∆ppk1 ∆ppk2B ∆ppk2C* *algP::algP-mApple*; deletion of PA14_69230, PA14_33240, and PA14_19410, replacement of *algP* (PA14_69370) with chimeric fusion *algP-mApple* | This study |

**Table S4b: Plasmids**

| Name | Genotype/Purpose |  | Source |
| --- | --- | --- | --- |
| pMQ30 | Suicide vector | Gm^R^ | (12) |
| pUC18R6K-  mini-Tn7T-Gm | Mobilizable mini-Tn7 base vector with MCS | Gm^R^ | (10) |
| PLREX9 | *ppk2a(*PA14_01730)*::ppk2a-mCherry*; pMQ30 derivative | Gm^R^ | (3) |
| pLREX62 | pUC18T-mini-Tn7T-G^R^ ParS^pMT1^ P_ssb_ *SSB-mCherry GFP-ParB ^pMT1^;* pUC18R6K-mini-Tn7T-Gm derivative | Gm^R^ | (3) |
| pLREX79 | *ppk2a(*PA14_01730)*::ppk2a-20aa-mNeonGreen*; pMQ30 derivative | Gm^R^ | This study |
| pLREX120 | *algP* (PA14_69370) deletion vector; pMQ30 derivative | Gm^R^ | This study |
| pLREX121 | *algP::algP*∆CTD; pMQ30 derivative, truncation of 195 c-terminal amino acids (157-352) of algP (PA14_69370) | Gm^R^ | This study |
| pLREX124 | *algP(*PA14_69370)*::mApple-algP*; pMQ30 derivative | Gm^R^ | This study |
| pLREX125 | *algP(*PA14_69370)*::algP-mApple*; pMQ30 derivative | Gm^R^ | This study |
| pLREX132 | pUC18T-mini-Tn7T-G^R^ *P_algP_:algP;* *algP* complementation, pUC18R6K-mini-Tn7T-Gm derivative | Gm^R^ | This study |

**Table S4c: Primers**

| Name | Purpose | Sequence | |
| --- | --- | --- | --- |
| LRPR894F | Construction of *algP* derivatives at native locus | | GACCATGATTACGAATTCGAGCTCGGTACCTCAGCGGACGCCCAGCAGGTCGATCTCG |
| LRPR912R | Construction of **pLREX120** *[∆algP*] | | CGCAGCCGGCTTGGCCGCAGGCTTCATGACGTGCCTCCAGGCGGACGTGGTTGCGCCC |
| LRPR909F | Construction of **pLREX120** *[∆algP*] | | GGCGCAACCACGTCCGCCTGGAGGCACGTCCTAAGGCGCTGTCTGCAAAGCCGCCGAGCC |
| LRPR899R | Construction of *algP* constructs at native locus | | CGTTGTAAAACGACGGCCAGTGCCAAGCTTATGACTGGAAATGTCTGGAAATTCGCGGTG |
| LRPR933R | Construction of **pLREX121** [*algP∆CTD*] | | GGCTCGGCGGCTTTGCAGACAGCGCCTTAGTTACGCCGCTACGGTTTTCATCGCAGGC |
| LRPR932F | Construction of **pLREX121** [*algP∆CTD*] | | GCCAAGCCTGCGATGAAAACCGTAGCGGCGTAACTAAGGCGCTGTCTGCAAAGCCGCCG |
| LRPR904R | Construction of **pLREX124** [*algP::mApple-algP*] | | GGCCATATTGTTTTCCTCGCCCTTCGACACCATGACGTGCCTCCAGGCGGACGTGGTTGC |
| LRPR905F | Construction of p **pLREX124** [*algP::mApple-algP*] | | GCAACCACGTCCGCCTGGAGGCACGTCATGGTGTCGAAGGGCGAGGAAAACAATATGGCC |
| LRPR906R | Construction of **pLREX124** [*algP::mApple-algP*] | | GGTGCAAGGGGGTGGTGACGGGCTTCTTGTTGGCCGATTTATAGAGTTCGTCCATCCCC |
| LRPR907R | Construction of **pLREX124** [*algP::mApple-algP*] | | GGGGATGGACGAACTCTATAAATCGGCCAACAAGAAGCCCGTCACCACCCCCTTGCACC |
| LRPR901R | Construction of **pLREX125** [*algP::algP-mApple*] | | GAACTCTTTGATGATGGCCATATTGTTTTCCTCGCCCTTCGACACGGAGGCGCTGGTCGGGGCGGCGCCGTTGCTGCCCG |
| LRPR900F | Construction of **pLREX125** [*algP::algP-mApple*] | | GCAACGGCGCCGCCCCGACCAGCGCCTCCGTGTCGAAGGGCGAGGAAAACAATATGGCC |
| LRPR903R | Construction of **pLREX125** [*algP::algP-mApple*] | | GCTTGGCTCGGCGGCTTTGCAGACAGCGCCTTAGTTATTTATAGAGTTCGTCCATCCCC |
| LRPR898F | Construction of **pLREX125** [*algP::algP-mApple*] | | GACGTGATGGGTATGGATGAACTCTATAAGTCGGCCAACAAGAAGCCCGTCACCACCC |
| LRPR956F | Construction of **pLREX132**  [*P_algP_:algP]* | | CTTATCTGGTTGGCCTGCAAGGCCTTCGCGAGGTACCGGGGCGGTTTCTCCAGACGAATC |
| LRPR955R | Construction of **pLREX132**  [*P_algP_:algP]* | | GTGGATCCCCCGGGCTGCAGGAATTCCTCGAGAAGCTTGGGTTAGGAGGCGCTGGTCGGG |
